# Disrupted brain functional networks in adolescents and young adults with gaming disorder during social interaction: An fNIRS study

**DOI:** 10.1017/S0033291726104176

**Published:** 2026-06-08

**Authors:** Zipan Wang, Chuanning Huang, Haidi Shan, Yue Wang, Shuo Li, Lei Guo, Xuechan Lyu, Yifu Chen, Yuhui Zeng, Hang Su, Tianzhen Chen, Jiang Du, Haifeng Jiang, Mengqiao Deng, Xifeng Wen, Min Zhao, Na Zhong

**Affiliations:** 1 Shanghai Jiao Tong University School of Medicine Affiliated Shanghai Mental Health, China; 2 School of Psychology, Shanghai Jiao Tong University, Shanghai, China; 3 Hunan Railway Professional Technology College, Mental Health Education and Counseling Center, China; 4 Third Hospital of Zhuzhou, China; 5 Shanghai Jiao Tong University Antai College of Economics and Management, China; 6 Shanghai Key Laboratory of Psychotic Disorders, Shanghai Mental Health Center, Shanghai, China

**Keywords:** brain networks, functional near-infrared spectroscopy, gaming disorder, prefrontal cortex, social interaction, temporoparietal junction

## Abstract

**Background:**

Gaming disorder (GD) is increasingly recognized for its adverse impact on social functioning, yet the underlying neural mechanisms remain unclear, particularly during real-time social interactions. This study examined neural differences in the prefrontal cortex (PFC) and temporoparietal junction (TPJ) during cooperation and competition tasks in adolescents and young adults with GD.

**Methods:**

Using functional near-infrared spectroscopy (fNIRS), we examined 175 male participants, classified into Gaming Disorder (n = 42), Hazardous Gaming (n = 69), and Healthy Control (n = 64) groups. Participants performed cooperation and competition tasks while activation, functional connectivity, and network topology were assessed within the PFC and TPJ.

**Results:**

In the current study, GD participants exhibited significantly greater impairments in social and emotional functioning compared to the HG and HC groups. The GD group showed increased right TPJ activation during cooperation and decreased activation during competition. Both GD and HG groups demonstrated heightened medial PFC activation and functional connectivity, indicating compensatory engagement. Graph theory analysis revealed disrupted network topology in GD, including reduced nodal efficiency and centrality within the PFC and TPJ. Neural alterations were significantly correlated with clinical measures, including gaming duration and social-emotional deficits.

**Conclusion:**

This study identifies critical neural disruptions underlying social dysfunction in GD, particularly within mentalizing and executive control networks. The neural markers observed in the GD group have important implications for clinical diagnosis and targeted intervention, whereas similar patterns in the HG group highlight opportunities for early detection and preventive strategies.

## Introduction

The rapid expansion of mobile internet technology and massively multiplayer online games (MMOGs) has dramatically reshaped the social and cognitive landscapes of young people. In response to growing concern, the World Health Organization included Gaming Disorder (GD) in the 11th revision of the International Classification of Diseases (ICD-11) as a distinct diagnosis (WHO, 2019), highlighting GD as an emerging public health challenge (Oka et al., 2021). Recent large-scale longitudinal cohort studies of Chinese adolescents further indicate that elevated or escalating Internet gaming disorder symptoms prospectively predict a range of adverse mental health outcomes, including increased depression, anxiety, sleep disturbances, and behavioral problems, as well as the onset, persistence and worsening of depressive symptoms, and suicidal ideation (Peng et al., 2025a, 2025b).

Among the most overlooked yet significant features of GD are impairments in social functioning, including increased social withdrawal, reduced empathy, heightened loneliness, and deteriorating peer relationships (Shoshani, Braverman, & Meirow, 2021; Yen, Lin, Wu, & Ko, 2022). These difficulties have been linked to both the development and persistence of problematic gaming (Mihara & Higuchi, 2017; Sioni, Burleson, & Bekerian, 2017). However, the neurobiological mechanisms that underlie disrupted social interaction in GD, particularly in real-time interpersonal contexts, remain insufficiently understood (Zheng et al., 2025). Accordingly, clarifying these neurobiological processes is essential for gaining a clearer understanding of GD-related social difficulties.

Neuroimaging studies have identified the prefrontal cortex (PFC) and temporoparietal junction (TPJ) as key regions supporting the social and regulatory processes required for successful interaction (Feng et al., 2021). The TPJ, particularly in the right hemisphere, is a key region of the mentalizing network and supports inferring others’ beliefs and intentions, embodied perspective-taking, and prosocial decision-making, thereby enabling flexible adaptation to social partners (Bitsch et al., 2018; Martin et al., 2020; Wang et al., 2016). The medial PFC (mPFC) contributes to mentalizing by integrating self-referential processing with self-other distinction, which is essential for interpreting complex interpersonal cues and maintaining coherent representations of social relationships (Finlayson-Short, Davey, & Harrison, 2020; Smallwood et al., 2021). Lateral PFC regions, including the dorsolateral (dlPFC) and ventrolateral (vlPFC) PFC, implement cognitive control operations such as goal maintenance, response inhibition, and top-down emotion regulation that allow behavior to be adjusted to social demands (Berboth & Morawetz, 2021; Mo et al., 2023; Zhao et al., 2021). Consistent with these roles, individuals with gaming disorder show structural and functional abnormalities within the TPJ and these prefrontal subregions, including altered activation and connectivity patterns linked to impulsivity, emotional dysregulation, and biased processing of social and game-related cues (Huang et al., 2024; Shin, Kim, Kim, & Kim, 2021; Zhang et al., 2020), suggesting that disruption of PFC–TPJ circuits may play a central role in the social dysfunction observed in GD.

Informed by prior evidence, the present study focuses on the PFC–TPJ circuitry to characterize neural dynamics during social interaction in GD. To capture interactive demands central to online gaming, we employed cooperative and competitive tasks, two fundamental modes of social engagement (Kingsbury & Hong, 2020). During cooperation, individuals coordinate their actions toward shared goals and continuously monitor a partner’s behavior to achieve joint outcomes, whereas during competition, they pursue conflicting goals, attempt to outperform an opponent, and evaluate their status relative to a rival (Qiao et al., 2025). Both interaction modes require inferring other’s intentions, anticipating behavior, and regulating one’s own responses in light of social feedback, engaging mentalizing, socio-emotional processing of success and failure, and higher-order cognitive control (Lee, Ahn, Kwon, & Kim, 2018). Compared with nonsocial paradigms that focus on isolated inhibition or reward processing, cooperation and competition more closely resemble the team-based play, rivalry, and social evaluation that characterize online gaming (Verheijen, Stoltz, van den Berg, & Cillessen, 2019). They, therefore, offer higher ecological validity for probing social dysfunction in GD. Given evidence that GD is also associated with broader cognitive difficulties, assessing cognitive performance alongside neural activity may help clarify individual differences that shape behavior in these interaction contexts (Wang et al., 2021).

Despite substantial progress in neuroimaging research on GD, several important gaps remain. Most studies have relied on MRI or fMRI methodologies, which restrict natural movement and are susceptible to motion artifacts, limiting the study of neural dynamics during realistic face-to-face interaction (Balachandrasekaran et al., 2021; Ciric et al., 2018). Additionally, task-based research has predominantly focused on reward processing and cue reactivity, with limited exploration of social dimensions (Li et al., 2020; Zheng et al., 2025). Notably, existing literature frequently contrasts GD individuals solely with healthy controls (Chen et al., 2020; Yan, Li, Yu, & Zhao, 2021), neglecting individuals engaged in Hazardous Gaming (HG), who may be on these moderate-risk trajectories and already exhibit emerging psychological and neural vulnerabilities (Griffiths, 2010; Peng et al., 2025c). Including an HG group enables early characterization of neural dysfunction, enhancing potential for early detection and intervention.

To address these gaps, the present study employed functional near-infrared spectroscopy (fNIRS), a neuroimaging technique characterized by high temporal resolution and ecological validity (Pinti et al., 2020; Pu et al., 2025), to examine cortical activation, functional connectivity, and brain network topology of PFC and TPJ regions, while adolescents and young adults with GD, HG, and healthy controls engaged in cooperative and competitive interaction. We hypothesized that (1) individuals with GD would show poorer performance in cooperation and competition tasks compared to healthy controls, with intermediate impairments observed in HG individuals; (2) during social interaction, GD would show decreased activation and functional connectivity in executive control regions (dlPFC, vlPFC), and increased activation and connectivity in social cognitive regions (TPJ, mPFC); and (3) GD would exhibit disrupted brain network topology, characterized by altered global and local connectivity metrics. Clarifying these mechanisms may improve understanding of social dysfunction in GD and inform the development of targeted early interventions.

## Methods

### Participants

From 1st November to 24th December, 2023, we recruited 175 subjects through online and offline advertisements in Zhuzhou, Hunan Province, China. All participants included were as follows: (1) right-handed, (2) age ≥12 years old, and (3) hearing/visual acuity or corrected-hearing/visual acuity in the normal range. Exclusion criteria were as follows: (1) diagnosed with other mental disorders under ICD-11 by senior psychiatrists or other diseases that affected cognitive function, such as a history of head trauma, cerebrovascular disease, and epilepsy; (2) use of cognitive-promoting drugs in the last 6 months; and (3) intellectual impairment, IQ < 70.

All participants who met the inclusion criteria and did not meet any exclusion criteria were subsequently evaluated by licensed psychiatrists. Based on clinical interviews and diagnostic assessments conducted in accordance with ICD-11 criteria, participants were classified into one of three groups: Gaming Disorder (GD), Hazardous Gaming (HG), or Healthy Controls (HC) (see Supplementary Methods, S1). The study was approved by the Ethics Committee of the Shanghai Mental Health Center (File number: 2023-63). All procedures complied with the Declaration of Helsinki. Written informed consent was obtained from all adult participants. For participants younger than 18 years, written informed consent was obtained from a parent or legal guardian, and assent was obtained from the adolescent participants.

### Scales and cognitive measurement

#### Scales

Assessment measures included demographic characteristics (e.g., age, gender), gaming-related behaviors (e.g., years of gaming experience, average daily gaming, game genre), gaming screening scales, emotion-related scales, social-related scales, and others.

Gaming disorder was screened using the Chinese version of the Gaming Disorder Screening Scale (GDSS; Lyu et al., 2022), and diagnoses were confirmed by qualified psychiatrists through structured clinical interviews based on the diagnostic criteria of the ICD-11. Emotional functioning was assessed using the Patient Health Questionnaire-9 (PHQ-9) for depressive symptoms (Costantini et al., 2021) and the Generalized Anxiety Disorder-7 (GAD-7) for anxiety symptoms (Kim & Lee, 2021). Relational self-esteem was measured with the Relational Self-Esteem Scale (RSES) (Winch, 1965), and impulsivity was assessed using the Barratt Impulsiveness Scale (BIS), including its cognitive, motor, and nonplanning subscales (Patton, Stanford, & Barratt, 1995). Social functioning was assessed with the UCLA Loneliness Scale-8 (ULS-8) for loneliness (Hays & DiMatteo, 1987), the Perceived Social Support Scale (PSSS) for perceived support (Zimet, Dahlem, Zimet, & Farley, 1987), the Lubben Social Network Scale-Revised (LSNS-R) for social network size and closeness (Lubben & Gironda, 2003), and the Social Interaction Anxiety Scale-6 (SIAS-6) for social anxiety (Mattick & Clarke, 1998) (see Supplementary Methods, S2).

#### Cognitive tasks

Participants completed computerized cognitive assessments via Cogstate (Mielke et al., 2014). In the present study, four tasks were selected to evaluate specific cognitive domains: Groton Maze Learning Test (GMLT) to assess spatial problem-solving ability, the Two-Back Test (TWB) to assess working memory, the Continuous Paired Associate Learning Task (CPAL) to assess spatial working memory, and the Social-Emotional Cognition Test (SECT) to assess emotion recognition (see Supplementary Methods, S3).

### Task design

**Figure 1.**
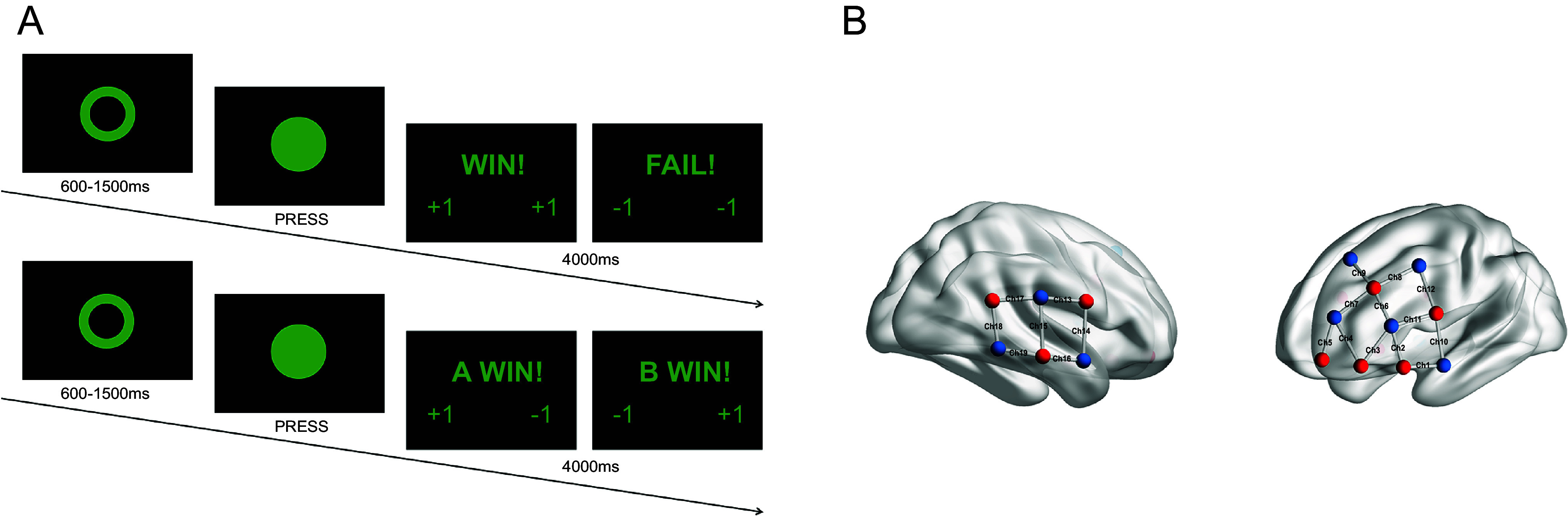
Task procedure and fNIRS optode configuration. (a) The procedure of each trial of the cooperation and competition tasks. (b) fNIRS optode layout. Red circles are sources, blue circles are detectors, and gray lines represent constructed channels between them. [All 3-D brain figures edited based on BrainNet Viewer (Xia, Wang, & He, 2013)]. 4 ROIs: medial prefrontal cortex: Ch6, Ch7, Ch8, Ch9, Ch12; left ventral lateral prefrontal cortex: Ch2, Ch3, Ch4, Ch5; left dorsolateral prefrontal cortex: Ch1, Ch10, Ch11; and right TPJ: Ch13, Ch14, Ch15, Ch16, Ch17, Ch18, Ch19. Figure 1. long description.

We used two independent, computer-based behavioral tasks, cooperation, and competition tasks, adapted from Cui, Bryant, and Reiss (2012) into a Chinese version (Figure 1a). Participants completed both tasks in dyads (pairs), while fNIRS signals were recorded. Each task comprised two blocks separated by a 30-s rest, and each block contained 20 trials. All analyses were conducted at the single-participant level (Cui, Bryant, & Reiss, 2012) (see Supplementary Methods, S4).

### fNIRS data acquisition

Task-related oxygenated hemoglobin (HbO) and deoxygenated hemoglobin (HbR) changes were measured using NIRSport2 (NIRx, Germany). The fNIRS system recorded at wavelengths of 760 nm and 850 nm, with a sampling rate of 10.1725 Hz. Sixteen optodes (8 sources × 8 detectors) formed 19 channels across four regions of interest (ROIs): medial prefrontal cortex (mPFC), left ventral lateral prefrontal cortex (LvlPFC), left dorsolateral prefrontal cortex (LdlPFC), and right temporoparietal junction (TPJ; Figure 1b).

### Data processing

#### Data preprocessing

Preprocessing utilized Homer2 in MATLAB (v2020b; Huppert, Diamond, Franceschini, & Boas, 2009). Raw data were converted to optical density, corrected for motion artifacts using MARA, and converted to HbO and HbR concentration changes via the modified Beer–Lambert law (PPF = 6.0). Motion artifacts were detected at the channel level using the Homer 2 function hmrMotionArtifactByChannel (tMotion = 1.0, tMask = 1.0, STDEVthresh = 20.0, AMPthresh = 5.0) (Cooper et al., 2012; Huang et al., 2024; Lu, Wang, Zhan, & Lu, 2025). To reduce the effect of high-frequency systemic physiological noise, such as respiration and heartbeat, and artifacts related to the very low frequency drift, we bandpass filtered signals in 0.01- to 0.09-Hz frequency band (Pinti et al., 2019). Supplementary material Table S1 reports, by group, the final N for behavioral and imaging analyses and the group-level average percentage of bad channels. HbO signals were analyzed due to their higher sensitivity and reliability compared to HbR (Fishburn, Norr, Medvedev, & Vaidya, 2014).

#### Brain areas activation

Mean baseline-corrected HbO activation (ΔHbO) was computed per channel during cooperation and competition tasks, relative to resting-state baselines.

#### Functional connectivity analysis

Pairwise functional connectivity was assessed using Pearson’s correlation coefficients calculated between filtered HbO time series, producing 19 × 19 matrices per participant per task. Values were Fisher Z-transformed to achieve normality (Fisher, 1915).

#### Construction of networks and graph theoretical analysis

Graph theoretical Network Analysis (GRETNA) toolbox was used for graph theory analysis (Wang et al., 2015). The nodes of the graph were defined as fNIRS measurement channels and the edges represented functional connection (Friston, 1994). Unweighted and undirected networks were constructed based on correlation (Vecchio et al., 2019). To ensure robustness, graph metrics were integrated across sparsity thresholds (0.1–0.4, step 0.1) using the area under curve (AUC) method (Liu et al., 2016; Gregorich, Simpson & Heinze, 2024).

We examined the topological properties of functional brain networks using four global and four nodal-level metrics. The global metrics included clustering coefficient (*C_p_*) and characteristic path length (*L_p_*), as well as global efficiency and local efficiency, which reflect the overall topological organization of the brain network. The nodal metrics were used to assess regional network characteristics and included nodal clustering coefficient, nodal efficiency, degree centrality, and betweenness centrality (Rubinov & Sporns, 2010).

#### Statistical analysis

Statistical analyses employed Python. Demographic and behavioral group differences were examined using ANOVA after verifying normality (Shapiro–Wilk test) and homogeneity of variance (Levene’s test). Pearson correlation analyses explored relationships between behavioral and fNIRS measures. ANOVA compared brain activation, connectivity, and network metrics across groups, with post hoc analyses using Tukey’s HSD tests. We controlled for multiple comparisons using the false discovery rate (FDR), with statistical significance defined as *p*_FDR_ < 0.05.

## Results

### Descriptive and behavioral results

All participants were male, with no significant differences in age among the three groups. Participants’ game-genre endorsements were recorded and are summarized by group as counts and percentages in Supplementary material Table S2. Compared to the HG and HC groups, individuals with GD showed significantly higher scores on the SIAS, PHQ-9, GAD-7, ULS, and RSES, as well as significantly lower scores in LSNS (*p* < 0.05), indicating more severe social anxiety, depression, generalized anxiety, loneliness, lower relational self-esteem, and poorer social interaction. No significant group differences were observed in BIS and PSSS scales (see Table 1).

**Table 1.** Demographic and clinical characteristics of GD, HG and HC Table 1. long description.

	GD*M (SD)*	HG*M (SD)*	HC*M (SD)*	*F*	*P*	*η^2^*
	N = 42	N = 67	N = 52			
Age	18.67(0.93)	18.85(1.00)	18.79(0.99)	F_(2,158)_ = 0.45	0.64	0.01
GDSS	53.10(7.67)	39.79(3.60)	20.23(3.83)	F_(2,158)_ = 512.20	<0.001	0.87
Gaming year	9.25(3.37)	7.51(2.45)	5.44(3.33)	F_(2,158)_ = 18.93	<0.001	0.19
Gaming days/week	6.25(1.55)	5.25(2.11)	2.92(2.15)	F_(2,158)_ = 35.94	<0.001	0.31
Gaming hours/day	5.07(2.92)	3.34(1.69)	2.22(1.54)	F_(2,158)_ = 22.54	<0.001	0.22
BIS	83.62(15.48)	85.88(10.62)	82.77(14.61)	F_(2,158)_ = 0.87	0.42	0.01
PSSS	57.33(14.81)	61.12(10.51)	59.48(10.95)	F_(2,158)_ = 1.17	0.32	0.02
LSNS	26.26(12.58)	33.08(9.90)	29.34(8.95)	F_(2,158)_ = 5.17	0.01	0.06
SAS	14.05(5.14)	9.44(4.06)	13.04(4.41)	F_(2,158)_ = 14.44	<0.001	0.15
PHQ	11.83(5.76)	2.00(3.36)	6.87(4.66)	F_(2,158)_ = 52.91	<0.001	0.40
GAD	8.21(5.56)	0.67(2.02)	4.12(3.78)	F_(2,158)_ = 43.15	<0.001	0.35
ULS	20.38(3.99)	13.42(4.21)	17.66(3.69)	F_(2,158)_ = 37.78	<0.001	0.32
RESE	25.64(4.25)	31.67(4.48)	28.63(4.43)	F_(2,158)_ = 21.93	<0.001	0.22

*Note:* GDSS = Gaming Disorder Screen Scale, BIS = Barratt Impulsiveness Scale, PSSS = Perceived Social Support Scale, LSNS = the Abbreviated Lubben Social Network Scale, SAS = Social Anxiety Scale, PHQ = Patient Health Questionnaire, GAD = Generalized Anxiety Disorder, ULS = UCLA Loneliness Scale, RSES = Rosenberg Self-esteem Scale.

Behavioral performance during the cooperation and competition tasks in the fNIRS paradigm was also analyzed. In the competition task, a significant main effect of group was observed for the mean reaction time difference (*F*_(2,169)_ = 3.80, *p* = 0.02, *η* ^2^ = 0.04), with post hoc analysis revealing a significant difference between the GD group (120.09 ± 105.89 ms) and the HC group (84.50 ± 45.25 ms). No other behavioral measures showed significant group differences (*ps* > 0.05, see Supplementary material Tables S3 and S4).

Behavioral performance on the Cogstate tasks is summarized in Supplementary material Table S5. A one-way ANOVA revealed a significant main effect of group for legal errors on the GMLT (GMLT_LER) (*F*_(2,145)_ = 4.04, *p* = 0.02, *η* ^2^ = 0.05). Post hoc comparisons indicated that the HG group (53.45 ± 12.94) made significantly more legal errors than the HC group (46.64 ± 15.26). No significant group differences were observed for performance on the TWB, CPAL, or SECT tasks (see Supplementary material Table S5).

### fNIRS results

#### Cortical activation

**Figure 2.**
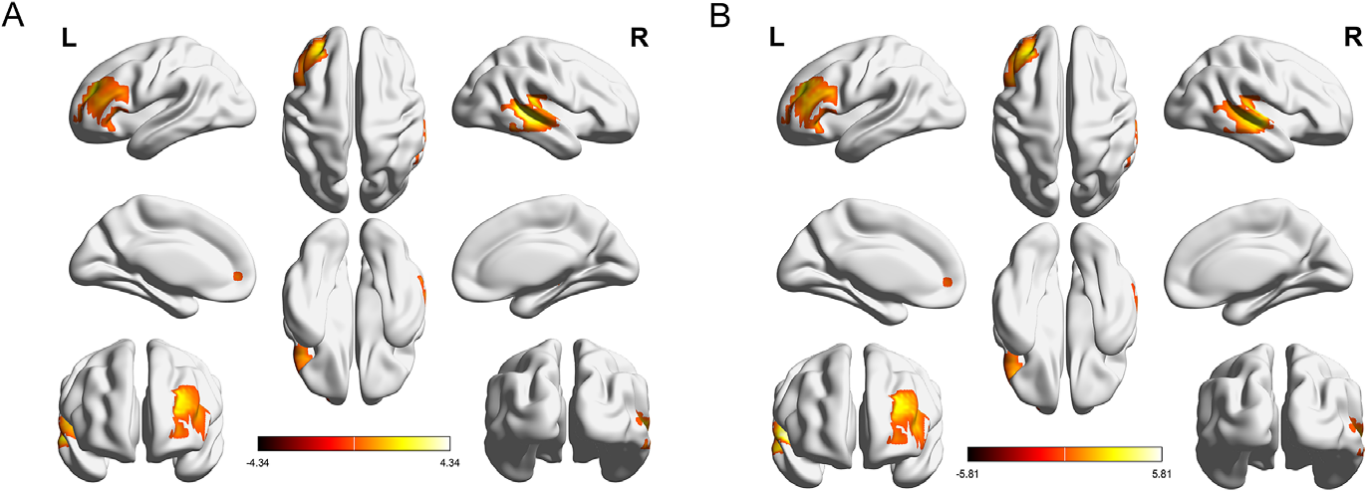
Significant group differences in brain activation among GD, HG, and HC groups. (a) Region in the RTPJ shows significant activation differences among the three groups during the cooperation task. (b) Region in the mPFC and RTPJ shows significant activation differences among the three groups in competition task. L, left hemisphere; R, right hemisphere. The color bar denoted the *F* value of contrast. Figure 2. long description.

In the cooperation task, a significant main effect of group was observed in RTPJ (*F*_(2,165)_ = 4.34, *p*_FDR_ = 0.02, *η* ^2^ = 0.05). Post hoc Tukey HSD analysis revealed that the GD group (0.68 ± 1.74) exhibited significantly greater activation compared to the HG group (−0.87 ± 3.22, *p*_FDR_ = 0.01) (see Figure 2a).

In the competition task, a significant main effect of group was found in mPFC (*F*_(2,157)_ = 3.64, *p*_FDR_ = 0.03, *η* ^2^ = 0.04). Post hoc comparisons indicated that the HG group (0.46 ± 1.77) exhibited significantly greater activation than the HC group (−0.34 ± 1.76, *p*_FDR_ = 0.03). Furthermore, a significant group effect was observed in RTPJ (*F*_(2,172)_ = 5.81, *p*_FDR_ = 0.004, *η* ^2^ = 0.06). Post hoc analyses revealed that the GD group (−0.58 ± 2.19) displayed significantly lower activation compared to both the HG group (0.65 ± 1.51, *p*_FDR_ = 0.003) and the HC group (0.44 ± 2.08, *p*_FDR_ = 0.02) (see Figure 2b).

#### Functional connectivity analysis

**Figure 3.**
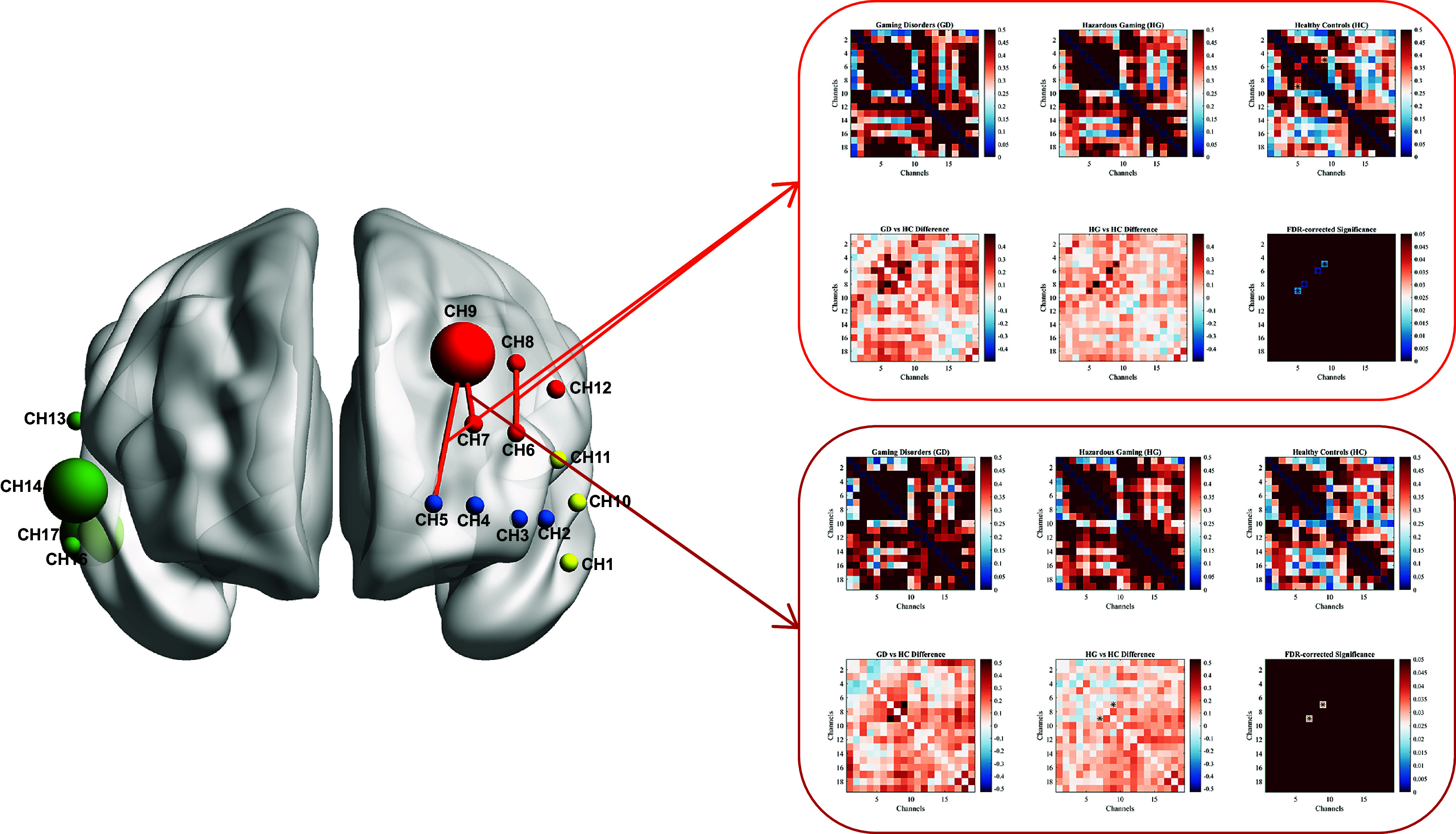
Group-level functional connectivity and intergroup differences during cooperation and competition tasks. The 3D brain rendering displays channels with significant activation (larger spheres) and significant functional connections (edges). Orange lines indicate significant connections during the cooperative task, while red lines represent those during the competitive task. The right panels show group-level functional connectivity (FC) matrices for the GD, HG, and HC groups, separately for cooperation (top) and competition (bottom) tasks. Corresponding group differences (GD vs. HC, HG vs. HC) and FDR-corrected significance maps (*p* < 0.05) are also displayed. Figure 3. long description.

The inter-group correlation matrices for channel connectivity are presented in Figure 3. In the cooperation task, a significant main effect of group was observed for functional connectivity between Channels 6 and 8 (*F*_(2, 103)_ = 12.25, *p*_FDR_ < 0.001, *η* ^2^ = 0.19). Post hoc Tukey HSD tests revealed that both the GD group (0.96 ± 0.42) and the HG group (0.98 ± 0.48) exhibited significantly stronger connectivity than the HC group (0.48 ± 0.54, *p*_FDR_ < 0.001). No significant difference was found between the GD and HG groups (*p*_FDR_ > 0.05).

Similarly, a significant main effect of group was identified for functional connectivity between Channels 5 and 9 (*F*_(2, 137)_ = 9.68, *p*_FDR_ = 0.001, *η* ^2^ = 0.12). Post hoc Tukey HSD tests indicated that both the GD group (0.75 ± 0.47) and the HG group (0.66 ± 0.55) showed significantly greater connectivity compared to the HC group (0.33 ± 0.46, *p*_FDR_ < 0.01). No significant difference was found between the GD and HG groups (*p*_FDR_ > 0.05).

In the competition task, a significant main effect of group was observed for functional connectivity between channels 7 and 9 (*F*_(2, 142)_ = 9.20, *p*_FDR_ < 0.001, *η* ^2^ = 0.11]. Post hoc Tukey HSD tests revealed that the GD group (1.16 ± 0.53) exhibited significantly stronger connectivity than both the HC group (0.63 ± 0.65) and the HG group (0.70 ± 0.59, *p*_FDR_ < 0.01). No significant difference was found between the HG and HC groups (*p*_FDR_ > 0.05) (see Figure 3).

#### Graph theory analysis

*Global network metrics.* One-way ANOVA revealed no significant main effect of group on the average clustering coefficient (*Cp*) in either the cooperation task (*F*_(2, 123)_ = 1.19, *p*_FDR_ = 0.31, *η* ^2^ = 0.02) or the competition task (*F*_(2, 123)_ = 1.20, *p*_FDR_ = 0.30, *η* ^2^ = 0.02). Characteristic path length (*Lp*) showed a significant main effect only during cooperation task (*F*_(2, 123)_ = 6.01, *p*_FDR_ = 0.003, *η* ^2^ = 0.09). Post hoc Tukey HSD tests indicated that the HC group had significantly shorter path lengths compared to the HG group (*MD* = −0.11, *p*_FDR_ = 0.04) and the GD group (MD = −0.15, *p*_FDR_ = 0.002). The group effect was not significant in competition task (*F*_(2, 123)_ = 1.95, *p*_FDR_ = 0.15, *η* ^2^ = 0.03).

Regarding network efficiency, the main effect of group was not statistically significant for either global efficiency (*E*_glob_) or local efficiency (*E*_loc_) in both tasks. For the cooperation task, *F*_(2, 123)_ = 1.70, *p*_FDR_ = 0.19, *η* ^2^ = 0.03 for *E*_glob_, and *F*_(2, 123)_ = 1.33, *p*_FDR_ = 0.27, *η* ^2^ = 0.02 for *E*_loc_. For the competition task, *F*_(2, 123)_ = 1.20, *p*_FDR_ = 0.31, *η* ^2^ = 0.02 for *E*_glob_, and *F*_(2, 123)_ = 1.49, *p*_FDR_ = 0.23, *η* ^2^ = 0.02 for *E*_loc_, indicating no statistically significant differences among the three groups.

**Figure 4.**
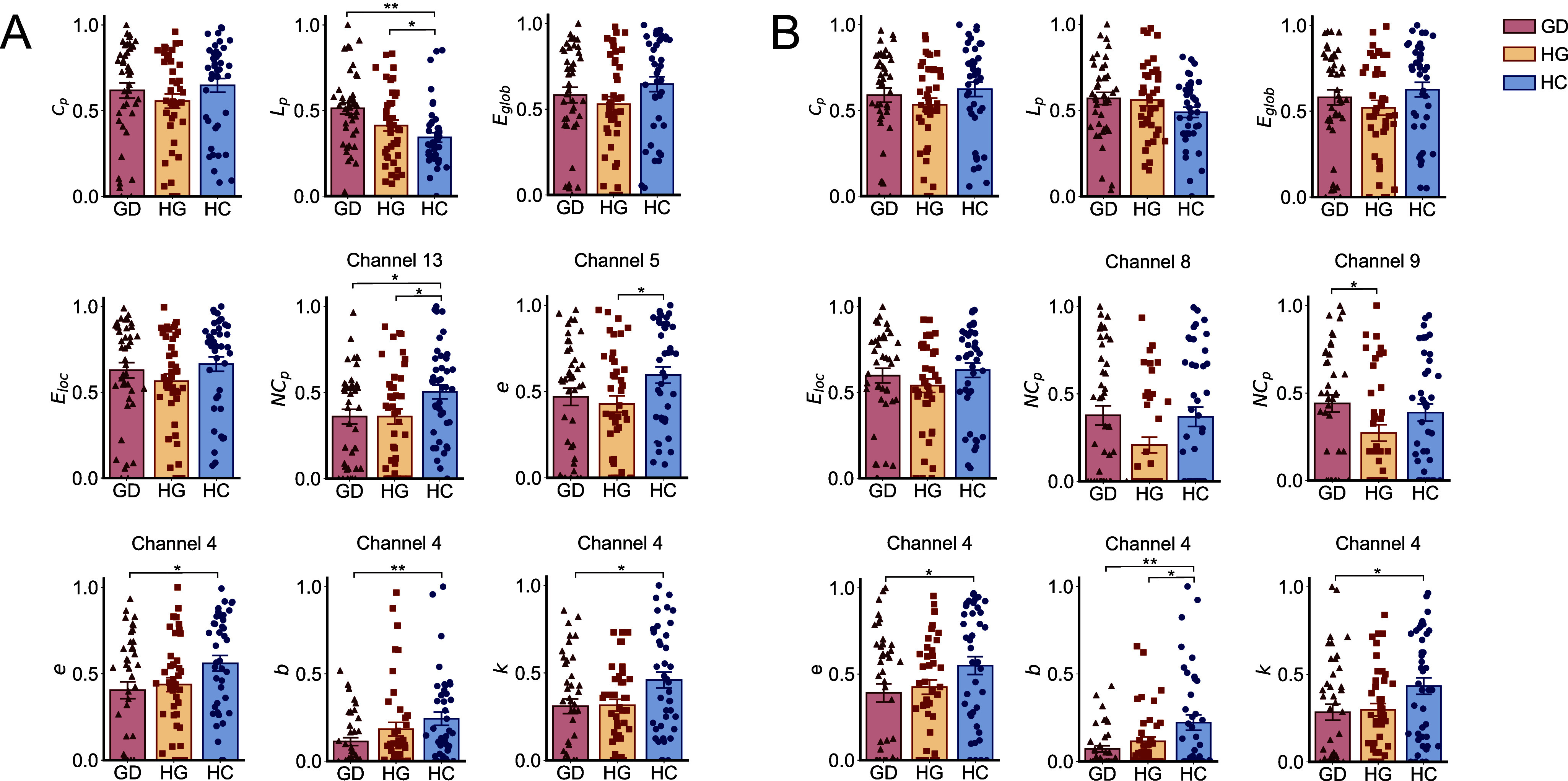
Group differences in global and nodal network metrics during cooperation and competition tasks. (a) Metrics during cooperation tasks. (b) Metrics during competition tasks. Global metrics: *Cp*, clustering coefficients; *Lp*, characteristic path length; *E*_glob_, global efficiency; *E*_loc_, local efficiency. Nodal metrics: *NCp*, nodal clustering coefficient; *e*, nodal efficiency; *b*, betweenness centrality; *k*, degree centrality. *p < 0.05, ^**^p < 0.01. The error bars indicate bootstrapped 95% confidence intervals. Figure 4. long description.

*Nodal network metrics.* During the cooperation task, the nodal clustering coefficient (*NCp*) at channel 13 showed a significant main effect of group (*F*_(2, 123)_ = 3.79, *p*_FDR_ = 0.03, *η* ^2^ = 0.06), with the HC group exhibiting significantly higher clustering coefficients compared to both the HG group (*MD* = 0.04, *p*_FDR_ = 0.05) and the GD group (*MD* = 0.04, *p*_FDR_ = 0.05). Nodal efficiency (*e*) at channel 4 also showed a significant group effect (*F*_(2, 123)_ = 3.78, *p*_FDR_ = 0.03, *η* ^2^ = 0.06), with the HC group demonstrating significantly higher efficiency than the GD group (*MD* = 0.03, *p*_FDR_ = 0.03). Furthermore, at channel 5 (*F*_(2, 123)_ = 3.65, *p*_FDR_ = 0.03, *η* ^2^ = 0.06), the HC group exhibited significantly higher efficiency than the HG group (*MD* = 0.03, *p*_FDR_ = 0.03). Regarding betweenness centrality (*b*), channel 4 showed a significant main effect of group (*F*_(2, 123)_ = 4.40, *p*_FDR_ = 0.01, *η* ^2^ = 0.07), with the HC group demonstrating significantly higher centrality than the GD group (*MD* = 1.42, *p*_FDR_ = 0.01). Degree centrality (*k*) followed the similar pattern (*F*_(2, 123)_ = 3.77, *p*_FDR_ = 0.03, *η* ^2^ = 0.06), with the HC group exhibiting significantly higher values than the GD group (*MD* = 0.42, *p*_FDR_ = 0.03) (see Figure 4a).

During the competition task, the nodal clustering coefficient (*NCp*) at channel 8 revealed a significant main effect of group (*F*_(2, 123)_ = 3.27, *p*_FDR_ = 0.04, *η* ^2^ = 0.05). At channel 9, a significant group effect was also found (*F*_(2, 123)_ = 3.10, *p*_FDR_ = 0.04, *η* ^2^ = 0.05), with post hoc comparisons indicating that the GD group had significantly higher clustering coefficients than the HG group (*MD* = 0.05, *p*_FDR_ = 0.04). Nodal efficiency (*e*) at channel 4 showed a significant effect (*F*_(2, 123)_ = 3.29, *p*_FDR_ = 0.04, *η* ^2^ = 0.05), with the HC group again demonstrating significantly higher efficiency than the GD group (*MD* = 0.03, *p*_FDR_ = 0.04). For betweenness centrality (*b*), channel 4 showed a significant main effect of group (*F*_(2, 123)_ = 6.21, *p*_FDR_ = 0.002, *η* ^2^ = 0.09), with the HC group showing significantly higher centrality than both the GD group (*MD* = 1.36, *p*_FDR_ = 0.004) and the HG group (*MD* = 1.23, *p*_FDR_ = 0.01). Degree centrality (*k*) at channel 4 also showed a significant group effect (*F*_(2, 123)_ = 3.59, *p*_FDR_ = 0.03, *η* ^2^ = 0.06), with the HC group exhibiting significantly higher values than the GD group (*MD* = 0.42, *p*_FDR_ = 0.04) (see Figure 4b). No significant task effects were observed at the neural level (see Supplementary material Tables S6 and S7).

### Correlation analysis

Correlation analysis between cortical activation, functional connectivity, and behavioral scale scores revealed that in the GD group, activation at channel 14 showed significant negative correlations with depressive symptoms (*r* = −0.62, *p*_FDR_ < 0.05). In the HG group, functional connectivity between channels 6 and 8 was positively correlated with gaming frequency (*r* = 0.46, *p*_FDR_ < 0.05) and with depressive symptoms (*r* = 0.52, *p*_FDR_ < 0.05) (see Supplementary material Figure S1).

## Discussion

To our knowledge, this study was the first to use fNIRS to uncover neural alterations during social interaction tasks in GD and HG individuals. Key findings revealed changes in cortical activation, functional connectivity, and network topology, particularly in the PFC and TPJ.

Clinically, individuals with GD scored significantly higher than HC on measures of social anxiety, loneliness, depression, and generalized anxiety and significantly lower on overall social interaction scores. These findings align with prior research indicating impaired social-emotional functioning and interpersonal relationships in individuals with GD (González-Bueso et al., 2018; Teng et al., 2021; Wang & Cheng, 2021). Behaviorally, the results provided partial support for Hypothesis 1. Specifically, the GD group exhibited significantly longer reaction time difference during competition, which may reflect impaired executive control, heightened emotional reactivity, or inefficient social-cognitive processing (Luijten et al., 2017; Shin, Kim, Kim, & Kim, 2021). However, no significant group differences were found in other behavioral measures, potentially due to task brevity and limited complexity (Choi et al., 2024).

At the neural level, the GD group exhibited heightened RTPJ activation during cooperation compared to HG, a pattern that was consistent with Hypothesis 2. Given the TPJ’s role in mentalizing and empathy, this hyperactivation likely represents compensatory engagement under excessive social cognitive demands (Ding et al., 2024; Tholen et al., 2020; Zhang et al., 2020). Contrary to expectation, during competition, GD participants showed significantly lower TPJ activation compared to both the HG and HC. Competitive contexts typically involve greater social threat and higher demands on perspective-taking, emotion regulation, and impulse control and, therefore, engage social-cognitive resources differently from cooperative contexts (Lee, Ahn, Kwon, & Kim, 2018; Lei & Rau, 2023). Within this framework, reduced RTPJ activation in GD may index impaired adaptive mentalizing, diminished sensitivity to others’ intentions, and a lack of motivation for adaptive social strategy adjustments (Ahmad et al., 2021; Zhang et al., 2017). Overall, the transition from TPJ hyperactivation in individuals with HG to TPJ hypoactivation in those with GD may represent a neurofunctional alteration that serves as a potential biomarker of progression along the gaming-related clinical continuum. Notably, decreased TPJ activation was associated with more severe depressive symptoms, suggesting that TPJ hypoactivation may capture cognitive-affective deficits central to pathological gaming behaviors.

Participants in the HG group demonstrated elevated medial prefrontal cortex (mPFC) activation during competition relative to HC. Hyperactivation in mPFC has previously been linked to craving, impulsivity, maladaptive reward evaluation, and disrupted cognitive control across various addictive disorders (Clark, Boileau, & Zack, 2019; Kim et al., 2021; Zhang et al., 2017). Enhanced mPFC activation under competitive pressure in HG individuals may reflect intensified self-focus, heightened reward sensitivity, and amplified socio-emotional reactivity, indicative of elevated cognitive load from social evaluative processes (Christian et al., 2024; DiMenichi & Tricomi, 2017).

Functional connectivity analyses provided additional insights into neural alterations underpinning social dysfunction in GD and HG. During cooperation, both the GD and HG groups exhibited stronger intra-mPFC connectivity than the HC group. Additionally, increased connectivity was observed between the vlPFC and mPFC. The mPFC is integral to self-referential processing, mentalizing, and decision-making (De Pisapia, Barchiesi, Jovicich, & Cattaneo, 2019; Meyer & Lieberman, 2018), while the vlPFC supports inhibitory control and emotion regulation (Yu et al., 2023). The heightened connectivity within these prefrontal regions likely reflects compensatory recruitment to manage the cognitive demands of social interaction, indicating inefficient resource allocation (Han et al., 2017). Typically, the default mode network (centered around mPFC) and executive control network (involving vlPFC) are negatively correlated (Geng et al., 2024). Hyperconnectivity between these networks has been documented in various neurodevelopmental and neuropsychiatric conditions, including autism (Anderson et al., 2011), Down syndrome (Anderson et al., 2013), and schizophrenia (Whitfield-Gabrieli et al., 2009). Overall, these connectivity patterns partially supported Hypothesis 2. In line with this, stronger prefrontal network connectivity in the HG group was correlated with higher gaming frequency and greater depressive symptomatology.

During competitive interactions, GD individuals also displayed stronger intra-mPFC connectivity compared to HG and HC groups, potentially reflecting intensified self-referential cognition, increased immersion in gaming roles, heightened reward craving, and maladaptive cognitive control under competitive pressure (Gerfo et al., 2019; Zhang et al., 2021). Prior studies have consistently associated excessive mPFC connectivity with craving, impulsivity, and distorted reward processing in addictive disorders (Kim et al., 2021; Yang et al., 2021). Thus, these findings underscore mPFC dysfunction as a crucial factor driving social maladaptation and compulsive gaming behaviors.

From a network-analytic perspective, characteristic path length differed across groups during the cooperation condition, indicating diminished network integration efficiency under socially collaborative demands in GD and HG (Park et al., 2017; Zhai et al., 2017). In contrast, the clustering coefficient and global and local efficiency showed no significant group differences. This pattern aligns with findings in other addictive disorders and may indicate that functional network alterations in GD are relatively subtle (Sjoerds et al., 2017; Wee et al., 2014). Regionally, the RTPJ in the GD group displayed decreased nodal clustering coefficients, indicating impaired local specialization and efficiency potentially underlying social-cognitive deficits (Bitsch et al., 2021). Furthermore, vlPFC demonstrated reduced nodal efficiency, betweenness centrality, and degree centrality, pointing to diminished emotional and social regulation (Chen et al., 2022). Taken together, these findings provided support for Hypothesis 3. Accordingly, targeted interventions addressing these specific neural alterations could be pivotal for early prevention and treatment.

Several limitations warrant consideration. First, fNIRS cannot access deep cortical or subcortical regions crucial for social cognition, necessitating future studies employing imaging modalities with higher spatial resolution. Second, given the higher prevalence of GD in males (Mihara & Higuchi, 2017), only male participants were included, which may limit the generalizability of the results. Future research should incorporate gender-diverse samples to elucidate sex-specific neural mechanisms in GD (Grace et al., 2021). Moreover, although all self-report scales showed acceptable internal consistency in our adolescent and young adult sample, most were originally developed for adults and age-related differences in measurement properties cannot be excluded. Finally, the cross-sectional design precludes causal inference regarding PFC and TPJ alterations in the development of GD (Zhou et al., 2019). Longitudinal studies are warranted to elucidate the causal pathways underlying these neural changes and their impact on the progression of GD.

## Conclusion

This study systematically investigated brain activation, functional connectivity, and network topology during social interaction in individuals with GD and HG using fNIRS, a method with high ecological validity. Results revealed abnormalities in activation and functional connectivity in key brain regions associated with executive control and mentalizing processes (PFC, TPJ), along with disrupted network topology. These neural abnormalities were significantly associated with indices of impaired social functioning, thereby delineating a clinically meaningful neurofunctional profile of social dysfunction in gaming-related populations. Task-evoked PFC–TPJ activation patterns and functional connectivity may represent candidate neural features. With further longitudinal and clinical validation, these fNIRS-derived indices could serve as auxiliary neurobiological markers that complement conventional clinical assessments when integrated with behavioral measures of social functioning. Such multimodal information may help clinicians conduct more fine-grained risk stratification, support early identification of individuals at elevated risk along the gaming-related clinical continuum, and provide objective, quantifiable endpoints for monitoring treatment response in GD and HG. Within this diagnostic and monitoring framework, the observed neurofunctional profiles also point to potential intervention directions. For example, social-cognitive and executive-control training, as well as noninvasive neuromodulatory approaches designed to strengthen functional integration between the PFC and TPJ, may be explored as individualized targets to reduce social-cognitive difficulties and improve everyday social functioning in individuals with problematic gaming.

## Supporting information

10.1017/S0033291726104176.sm001 Wang et al. supplementary material 1 Wang et al. supplementary material

10.1017/S0033291726104176.sm002 Wang et al. supplementary material 2 Wang et al. supplementary material

## Data Availability

The data that support the findings of this study are available from the corresponding author upon reasonable request.

## Supplementary material

The supplementary material for this article can be found at http://doi.org/10.1017/S0033291726104176.

## Figure 1. Long description

Panel A contains two horizontal sequences. The top sequence shows a hollow green circle, then a filled green circle labeled PRESS, followed by two black screens labeled WIN plus one plus one and FAIL minus one minus one, each displayed for four thousand milliseconds. The bottom sequence starts with the same hollow and filled green circles, then PRESS, followed by two screens labeled A WIN plus one minus one and B WIN minus one plus one, also for four thousand milliseconds. Both sequences have time intervals of six hundred to fifteen hundred milliseconds before PRESS. Panel B displays two three-dimensional brain renderings. The left brain shows red circles labeled as sources and blue circles as detectors, connected by gray lines labeled Ch1, Ch2, Ch3, Ch4, Ch5, Ch10, Ch11. The right brain shows additional channels Ch6, Ch7, Ch8, Ch9, Ch12, Ch13, Ch14, Ch15, Ch16, Ch17, Ch18, Ch19. Four regions of interest are indicated: medial prefrontal cortex (Ch6, Ch7, Ch8, Ch9, Ch12), left ventral lateral prefrontal cortex (Ch2, Ch3, Ch4, Ch5), left dorsolateral prefrontal cortex (Ch1, Ch10, Ch11), and right T P J (Ch13 to Ch19). All brain renderings are based on BrainNet Viewer.

Navigate back to Figure 1..

## Table 1. Long description

From top to bottom, the table lists variables in the first column: Age, GDSS, Gaming year, Gaming days per week, Gaming hours per day, BIS, PSSS, LSNS, SAS, PHQ, GAD, ULS, and RESE. For each variable, the next three columns show mean and standard deviation for GD (N equals 42), HG (N equals 67), and HC (N equals 52). The following columns display F statistic, P value, and eta squared. For Age, means are 18.67 (0.93), 18.85 (1.00), and 18.79 (0.99) with F sub 2,158 equals 0.45, P equals 0.64, eta squared equals 0.01. For GDSS, means are 53.10 (7.67), 39.79 (3.60), 20.23 (3.83), F sub 2,158 equals 512.20, P less than 0.001, eta squared equals 0.87. Gaming year: 9.25 (3.37), 7.51 (2.45), 5.44 (3.33), F sub 2,158 equals 18.93, P less than 0.001, eta squared equals 0.19. Gaming days per week: 6.25 (1.55), 5.25 (2.11), 2.92 (2.15), F sub 2,158 equals 35.94, P less than 0.001, eta squared equals 0.31. Gaming hours per day: 5.07 (2.92), 3.34 (1.69), 2.22 (1.54), F sub 2,158 equals 22.54, P less than 0.001, eta squared equals 0.22. BIS: 83.62 (15.48), 85.88 (10.62), 82.77 (14.61), F sub 2,158 equals 0.87, P equals 0.42, eta squared equals 0.01. PSSS: 57.33 (14.81), 61.12 (10.51), 59.48 (10.95), F sub 2,158 equals 1.17, P equals 0.32, eta squared equals 0.02. LSNS: 26.26 (12.58), 33.08 (9.90), 29.34 (8.95), F sub 2,158 equals 5.17, P equals 0.01, eta squared equals 0.06. SAS: 14.05 (5.14), 9.44 (4.06), 13.04 (4.41), F sub 2,158 equals 14.44, P less than 0.001, eta squared equals 0.15. PHQ: 11.83 (5.76), 2.00 (3.36), 6.87 (4.66), F sub 2,158 equals 52.91, P less than 0.001, eta squared equals 0.40. GAD: 8.21 (5.56), 0.67 (2.02), 4.12 (3.78), F sub 2,158 equals 43.15, P less than 0.001, eta squared equals 0.35. ULS: 20.38 (3.99), 13.42 (4.21), 17.66 (3.69), F sub 2,158 equals 37.78, P less than 0.001, eta squared equals 0.32. RESE: 25.64 (4.25), 31.67 (4.48), 28.63 (4.43), F sub 2,158 equals 21.93, P less than 0.001, eta squared equals 0.22. Notes define all abbreviations: GDSS is Gaming Disorder Screen Scale, BIS is Barratt Impulsiveness Scale, PSSS is Perceived Social Support Scale, LSNS is Abbreviated Lubben Social Network Scale, SAS is Social Anxiety Scale, PHQ is Patient Health Questionnaire, GAD is Generalized Anxiety Disorder, ULS is U C L A Loneliness Scale, RESE is Rosenberg Self-esteem Scale.

Navigate back to Table 1..

## Figure 2. Long description

Panel A on the left displays six views of a 3D-rendered brain, with L and R labels marking left and right hemispheres. The top row shows lateral views, the middle row shows medial views, and the bottom row shows dorsal views. Orange to yellow overlays highlight regions in the right temporoparietal junction and left hemisphere, indicating significant activation differences among groups during the cooperation task. Below, a horizontal color bar ranges from negative four point three four (dark red) to positive four point three four (yellow-white), labeled F value of contrast. Panel B on the right mirrors the arrangement, with overlays in the medial prefrontal cortex and right temporoparietal junction during the competition task. Its color bar ranges from negative five point eight one to positive five point eight one. Both panels use identical spatial orientation and color mapping to compare activation patterns across tasks and groups.

Navigate back to Figure 2..

## Figure 3. Long description

At the center, a 3D rendering of a brain displays spheres labeled C H 1 to C H 17. Spheres vary in size and color: large red spheres at C H 8 and C H 9, green at C H 13 and C H 14, blue at C H 3 to C H 5, and yellow at C H 1, C H 10, and C H 11. Orange lines connect red spheres, indicating significant connections during cooperation, while a red line links C H 8 to C H 10 for competition. Two orange arrows extend rightward to two rectangular panels. The upper panel contains six square matrices: the top row shows group-level functional connectivity for Giving Disorder (G D), Hoarding (H G), and Healthy Controls (H C) during cooperation, each matrix labeled on the top edge, with axes labeled Channels and color bars indicating connectivity strength. The bottom row shows G D versus H C difference, H G versus H C difference, and F D R-corrected significance, with the last matrix highlighting significant channel pairs. The lower panel repeats this structure for the competition task, with corresponding group matrices and difference maps. Each matrix uses a red-blue color scale, with red for higher connectivity and blue for lower. The spatial arrangement emphasizes the mapping between brain regions and connectivity patterns across groups and tasks.

Navigate back to Figure 3..

## Figure 4. Long description

Panel A on the left shows nine bar graphs for cooperation tasks. The top row displays C sub p, L sub p, and E glob, with L sub p and E glob showing significant differences between groups, indicated by single and double asterisks. The middle row shows E loc, N C sub p for Channel 13, and e for Channel 5, with N C sub p and e showing significant group differences. The bottom row shows e, b, and k for Channel 4, with e and b showing significant differences. Panel B on the right shows nine bar graphs for competition tasks. The top row displays C sub p, L sub p, and E glob, with no significant differences. The middle row shows E loc, N C sub p for Channel 8, and N C sub p for Channel 9, with N C sub p for Channel 9 showing a significant difference. The bottom row shows e, b, and k for Channel 4, with b showing a significant difference. All y-axes range from 0 to 1. GD bars are pink, HG bars are orange, and HC bars are blue. Error bars represent bootstrapped 95 percent confidence intervals. Statistical significance is indicated by a single asterisk for p less than 0.05 and double asterisks for p less than 0.01.

Navigate back to Figure 4..
