# Supplementary material for: Disrupted brain functional networks in adolescents and young adults with gaming disorder during social interaction: An fNIRS study
Source: Psychol Med. 2026 Jun 8;56:e189. doi: 10.1017/S0033291726104176 (PMC13247798; doi:10.1017/S0033291726104176)
Supplement: Wang et al. supplementary material 1 — Wang et al. supplementary material [file S0033291726104176sup001.docx]

**Supplementary Materials for**

**Disrupted Brain Functional Networks in Adolescents and Young Adults with Gaming Disorder During Social Interaction: An fNIRS Study**

Zipan Wang^1,2#^, Chuanning Huang^1#^, Haidi Shan^1^, Yue Wang^1^, Shuo Li^1,2^, Lei Guo^1^, Xuechan Lyu^1^, Yifu Chen^4^, Yuhui Zeng^3^, Hang Su^1^, Tianzhen Chen^1^, Jiang Du^1^, Haifeng Jiang^1^, Mengqiao Deng^5^, Xifeng Wen^5^, Min Zhao^1,6*^, Na Zhong^1*^

1 Shanghai Mental Health Centre, Shanghai Jiao Tong University School of Medicine, Shanghai, China

2 Shanghai Jiao Tong University School of Psychology, Shanghai, China

3 The Third Hospital of Zhuzhou, Hunan, China

4 Hunan Railway Professional Technology College, Mental Health Education and Counseling Center, Hunan, China

5 Antai College of Economics & Management, Shanghai Jiao Tong University, Shanghai, China

6 Shanghai Key Laboratory of Psychotic Disorders, Shanghai Mental Health Center, Shanghai, China

^#^ Equal contribution.

****Correspondence to***: winco917@hotmail.com (N. Zhong), drminzhao@smhc.org.cn (M. Zhao).

**Supplementary Methods**

**S1 Participants and diagnostic procedures**

Group allocation was based on the Chinese version of the Gaming Disorder Screening Scale (GDSS; Lyu et al., 2022) and ICD-11 clinical interviews. Gaming disorder (GD) was diagnosed when participants met ICD-11 criteria for gaming disorder at the structured clinical interview. Hazardous gaming (HG) followed the ICD-11 category of ‘hazardous gaming’, defined as a problematic pattern of gaming that clearly increases the risk of physical or mental harm to the individual or others, and requires monitoring or intervention, but does not yet meet the diagnostic requirements for gaming disorder. Operationally, HG cases in this study were those who showed subthreshold GDSS symptoms together with clinician-rated functional risk or impairment related to gaming, but did not meet full ICD-11 criteria for gaming disorder. Healthy controls (HC) did not meet ICD-11 criteria for either gaming disorder or hazardous gaming and reported no clinically significant gaming-related functional impairment or risk at interview.

**S2 Scales**

**Gaming Disorder Screening Scale (GDSS)**

Gaming disorder symptoms were assessed using the Chinese version of the Gaming Disorder Screening Scale (GDSS; Lyu et al., 2022), which comprises 18 items aligned with the diagnostic criteria of both DSM-5 and ICD-11. Each item is rated on a 4-point Likert scale (1 = never, 2 = sometimes, 3 = often, 4 = always), yielding a total score ranging from 18 to 72. The Cronbach's alpha coefficient for internal consistency reliability in the present study was 0.96.

**Patient Health Questionnaire-9 (PHQ-9)**

Depressive symptoms were evaluated using the PHQ-9 (Costantini et al., 2021; Levis et al., 2019), which consists of 9 items rated on a 4-point scale, with total scores ranging from 0 to 27. Higher scores reflect greater severity of depressive symptoms. Standard cutoff scores of 5, 10, 15, and 20 indicate mild, moderate, moderately severe, and severe depression, respectively. The Cronbach's alpha coefficient for internal consistency reliability in the present study was 0.93.

**General Anxiety Disorder-7 (GAD-7)**

Anxiety symptoms over the past two weeks were assessed using the GAD-7 (Löwe et al., 2008), which includes 7 items rated on a 4-point scale (0 = not at all, 1 = several days, 2 = more than half the days, 3 = nearly every day). Total scores range from 0 to 21, with thresholds of 5, 10, and 15 indicating mild, moderate, and severe anxiety, respectively. A score ≥ 10 is commonly used as the clinical cutoff for generalized anxiety disorder. The Cronbach's alpha coefficient for internal consistency reliability in the present study was 0.96.

**Relational Self-Esteem Scale (RSES)**

The RSES comprises 10 items designed to measure self-esteem derived from close interpersonal relationships (Winch, 1965). Five items are positively worded and five are negatively worded (Items 3, 5, 8, 9, and 10 are reverse-scored). Higher scores reflect greater levels of relational self-esteem. The Cronbach's alpha coefficient for internal consistency reliability in the present study was 0.80.

**Barratt impulsiveness scale (BIS-11)**

The BIS-11 (Li et al., 2011) includes 30 items covering three dimensions of impulsivity: motor impulsiveness, cognitive impulsiveness, and non-planning impulsiveness. Items are rated on a 5-point Likert scale (1 = rarely/never, 2 = occasionally, 3 = sometimes, 4 = often, 5 = almost always/always). Higher total scores indicate greater impulsivity. The Cronbach's alpha coefficient for internal consistency reliability of the overall scale was 0.88, with the reliability for the three dimensions being 0.79, 0.88, and 0.86, respectively.

**UCLA Loneliness Scale-8 (ULS-8)**

The ULS-8 (Hays & DiMatteo, 1987) is a short version of the UCLA Loneliness Scale, consisting of 8 items-6 positively worded (indicating loneliness) and 2 negatively worded (reverse scored). Items are rated on a 4-point scale (1 = never, 2 = rarely, 3 = sometimes, 4 = always), with total scores ranging from 8 to 32. Higher scores indicate greater perceived loneliness. The Cronbach's alpha coefficient for internal consistency reliability in the present study was 0.81.

**Perceived Social Support Scale (PSSS)**

The Perceived Social Support Scale (PSSS) (Zimet, Dahlem, Zimet & Farley, 1987) is designed to assess individuals’ perceived availability of social support from various sources, including family members, friends, colleagues, or classmates. It consists of 12 self-report items covering two subscales: support from within the family and support from outside the family. Each item is rated on a 7-point Likert scale ranging from 1 (strongly disagree) to 7 (strongly agree), with higher scores indicating higher perceived social support. The Cronbach's alpha coefficient for internal consistency reliability of the overall scale was 0.94, with the reliability for the two dimensions being 0.82 and 0.93, respectively.

**Lubben Social Network Scale-Revised (LSNS-R)**

The LSNS-R (Lubben & Gironda, 2003) assesses the respondent’s social engagement with both family and friends, capturing the size, closeness, and contact frequency of active social networks from which support is perceived (Lubben & Gironda, 2003). It comprises 12 self-report items divided into two subscales—family and friends. Items are rated on a five-point Likert scale (0-5), yielding total scores from 0 to 60; higher scores indicate greater social connectedness. The Cronbach's alpha coefficient for internal consistency reliability of the overall scale was 0.89, with the reliability for the two dimensions being 0.82 and 0.87, respectively.

**Social Interaction Anxiety Scale (SIAS-6)**

The SIAS-6 (Mattick & Clarke, 1998) is a brief measure of social interaction anxiety, consisting of 6 items. Participants rate the degree to which each item describes them on a 5-point scale: 0 = not at all characteristic of me, 1 = slightly uncharacteristic, 2 = neutral/unsure, 3 = somewhat characteristic, 4 = extremely characteristic. Higher scores indicate greater levels of social interaction anxiety. The Cronbach's alpha coefficient for internal consistency reliability in the present study was 0.82.

Instruments have prior support in adolescent and young-adult cohorts (Alorani & Alradaydeh, 2018; Du, King, & Chi, 2012; Ip et al., 2022; Lyu et al., 2022; Levis et al., 2019; Novotny et al., 2010; Vasconcelos, Malloy-Diniz, & Correa, 2012; Song et al., 2024; Watabe et al., 2015; Wu & Yao, 2008; Yildiz & Duy, 2014; Zsido, Varadi-Borbas, & Arato, 2021).

**S3 Cognitive tasks**

***Groton Maze Learning Test (GMLT)***

Participants were instructed to discover a hidden maze path by sequentially selecting tiles to reach a designated goal tile. This task served as a measure of executive function, with task duration used as the primary outcome variable.

***Two Back Test (TWB)***

Participants viewed a continuous sequence of playing cards and were required to indicate whether the current card matched the one presented two trials earlier in both suit and number. This task assessed working memory, with response speed as the outcome measure.

***Continuous Paired Associate Learning Task (CPAL)***

Participants were shown a central stimulus and asked to select the correct surrounding image based on previously learned color-shape associations and sequence. This task evaluated spatial working memory, with accuracy as the performance metric.

***Social-Emotional Cognition Test (SECT)***

Participants were presented with four facial expressions and instructed to identify the one that differed emotionally from the other three. This task assessed emotion recognition and social-perceptual processing, with accuracy used as the primary indicator.

**S4 Task design**

During fNIRS acquisition, we used two independent computer based tasks, cooperation and competition, adapted from Cui, Bryant, and Reiss (2012). Each task comprised two blocks separated by a 30 s rest, and each block contained 20 trials. At the single trial level, each trial included a ready cue, a go signal that initiated an approximately 0.6 to 1.5 s response window, a 4 s feedback screen, and a 2 s intertrial interval. Task order was fixed, with cooperation completed first and competition completed second.

In the cooperation task, subjects in pairs were asked to press the button together once the green hollow circle on the screen turned into solid. They could both get one point when they reacted as closely as possible. Otherwise, each of them lost one point. In the competition task, each subject needed to press the button as quickly as possible. Whoever reacted more quickly would get one point (Cui, Bryant, & Reiss, 2012).

**Table S1. Sample disposition and channel quality by analysis tier and group**

| **Analysis Tier** | **Group** | **Final**  **N** | **Excluded**  **(attention/random)** | **Avg. Bad-Channel % (SD)** |
| --- | --- | --- | --- | --- |
| Behavioral | HC | 52 | 12 | – |
|  | HG | 67 | 2 | – |
|  | GD | 42 | 0 | – |
| Imaging (fNIRS) | HC | 64 | – | 11.18% (11.62%) |
|  | HG | 69 | – | 9.38% (12.54%) |
|  | GD | 42 | – | 7.02% (8.98%) |

*Note.* GD, gaming disorder; HG, hazardous gaming; HC, healthy control.

**Supplementary Results**

**Table S2. Prevalence of primary game genres by group (n, %)**

| **Genre** | **ALL** | **GD** | **HG** | **HC** | **Test** | **Statistic** | ***p*** | ***p_fdr*** |
| --- | --- | --- | --- | --- | --- | --- | --- | --- |
|  | **N=161** | **N=42** | **N=67** | **N=52** |  | **(*df*)** |  |  |
| MOC | 105 | 34 (81.0%) | 43 (64.2%) | 28 (53.8%) | Chi-square | 7.580 (2) | 0.02 | 0.18 |
| RPG | 4 | 1 (2.4%) | 3 (4.5%) | 0 (0.0%) | Fisher | — | 0.37 | 0.55 |
| FPS | 44 | 10 (23.8%) | 22 (32.8%) | 12 (23.1%) | Chi-square | 1.758 (2) | 0.42 | 0.55 |
| BCG | 9 | 3 (7.1%) | 4 (6.0%) | 2 (3.8%) | Fisher | — | 0.83 | 0.90 |
| CMS | 13 | 5 (11.9%) | 6 (9.0%) | 2 (3.8%) | Fisher | — | 0.38 | 0.55 |
| CAS | 5 | 3 (7.1%) | 1 (1.5%) | 1 (1.9%) | Fisher | — | 0.27 | 0.55 |
| SPR | 8 | 2 (4.8%) | 4 (6.0%) | 2 (3.8%) | Fisher | — | 0.90 | 0.90 |
| OTH | 29 | 11 (26.2%) | 9 (13.4%) | 9 (17.3%) | Chi-square | 2.871 (2) | 0.24 | 0.55 |

*Notes.* Values are n (%) of participants within group endorsing each genre. Because genres were multi-label, column percentages do not sum to 100%. Tests compare endorsement rates across groups (3×2 tables). Pearson’s χ²used when Cochran’s assumptions were met; otherwise Fisher’s exact test. Multiplicity was controlled across the eight genre tests using FDR. **p*<0.05, ***p*<0.01, ****p*<0.001.

Abbreviations: HC = Healthy Controls; HG = Hazardous Gaming; GD = Gaming Disorder. MOC = Multiplayer Online Competitive; RPG = Role-Playing Games; FPS = Shooter (first-/third-person); BCG = Board-Card/ Strategy; CMS = Construction & Management Simulation; CAS = Casual games; SPR = Sports & Racing; OTH = Other.

**Table S3.** Behavioral Performance on the Cooperation Task

Across GD, HG, and HC Groups

|  | GD*M (SD)* | HG*M (SD)* | HC*M (SD)* | *F* | *P* | *η*² |
| --- | --- | --- | --- | --- | --- | --- |
|  | N=42 | N=65 | N=64 |  |  |  |
| Mean RT (ms) | 343.05(158.20) | 312.91(111.49) | 307.43(98.19) | F_(2,168)_ = 1.22 | 0.30 | 0.01 |
| Mean RT difference (ms) | 105.76 (74.85) | 121.48(114.06) | 117.58(95.53) | F_(2,168)_ = 0.34 | 0.72 | 0.004 |
| Score | 110.62 (11.03) | 113.14 (10.65) | 112.00 (9.93) | F_(2,168)_ = 0.74 | 0.48 | 0.01 |
| Successful Trials | 25.31 (5.52) | 26.57 (5.32) | 26.00 (4.97) | F_(2,168)_ = 0.74 | 0.48 | 0.01 |
| Failed Trials | 14.69 (5.52) | 13.43 (5.32) | 14.00 (4.97) | F_(2,168)_ = 0.74 | 0.48 | 0.01 |

**Table S4.** Behavioral Performance on the Competition Task

Across GD, HG, and HC Groups

|  | GD*M (SD)* | HG*M (SD)* | HC*M (SD)* | *F* | *P* | *η*² |
| --- | --- | --- | --- | --- | --- | --- |
|  | N=42 | N=67 | N=63 |  |  |  |
| Mean RT (ms) | 274.97(77.08) | 256.81(49.48) | 257.74(55.17) | F_(2,169)_ = 1.42 | 0.24 | 0.02 |
| Mean RT difference (ms) | 120.09 (105.89) | 93.73(47.16) | 84.50(45.25) | F_(2,169)_ = 3.80 | 0.02^*^ | 0.04 |
| Successful Trials | 20.71(7.12) | 19.13(5.95) | 20.08(6.94) | F_(2,169)_ = 0.79 | 0.46 | 0.01 |
| Score | 101.43(14.23) | 98.27(11.90) | 100.16(13.89) | F_(2,169)_= 0.79 | 0.46 | 0.01 |

*Note.* GD, gaming disorder; HG, hazardous gaming; HC, healthy control; ^*^*p* < 0.05, ^**^*p* < 0.01.

**Table S5.** Behavioral Performance on Cogstate Task

Across GD, HG, and HC Groups

|  | GD*M (SD)* | HG*M (SD)* | HC*M (SD)* | *F* | *P* | *η*² |
| --- | --- | --- | --- | --- | --- | --- |
|  | N=36 | N=62 | N=50 |  |  |  |
| GMLT_LER | 53.16(12.07) | 53.45(12.94) | 46.64(15.26) | F_(2,145)_ = 4.04 | 0.02^*^ | 0.05 |
| TWB_LMN | 2.97(0.14) | 2.99(0.15) | 3.00(0.12) | F_(2,145)_ = 0.34 | 0.71 | 0.01 |
| CPAL_ACC | 0.88(0.24) | 0.97(0.23) | 0.96(0.27) | F_(2,145)_ = 1.53 | 0.22 | 0.02 |
| SECT_ACC | 0.87(0.23) | 0.95(0.23) | 0.88(0.25) | F_(2,145)_= 1.72 | 0.18 | 0.02 |

*Note.* LER, legal errors; LMN, speed; ACC, accuracy; ^*^*p* < 0.05.

**Table S6.** FDR corrected *p* values for group and condition effects

on channel cortical activation

| **Channel** | **Group_*P*** | **Group_*P*_fdr_** | **Condition_*P*** | **Condition_*P*_fdr_** | **Interaction_*P*** | **Interaction_*P*_fdr_** |
| --- | --- | --- | --- | --- | --- | --- |
| 1 | 0.408 | 0.604 | 0.229 | 0.738 | 0.998 | 0.998 |
| 2 | 0.230 | 0.437 | 0.753 | 0.795 | 0.855 | 0.956 |
| 3 | 0.200 | 0.422 | 0.206 | 0.738 | 0.159 | 0.432 |
| 4 | 0.672 | 0.798 | 0.420 | 0.738 | 0.657 | 0.892 |
| 5 | 0.104 | 0.422 | 0.553 | 0.738 | 0.814 | 0.956 |
| 6 | 0.072 | 0.422 | 0.869 | 0.869 | 0.489 | 0.715 |
| 7 | 0.111 | 0.422 | 0.125 | 0.738 | 0.352 | 0.557 |
| 8 | 0.136 | 0.422 | 0.631 | 0.738 | 0.032 | 0.203 |
| 9 | 0.016 | 0.304 | 0.614 | 0.738 | 0.343 | 0.557 |
| 10 | 0.413 | 0.604 | 0.161 | 0.738 | 0.758 | 0.956 |
| 11 | 0.514 | 0.651 | 0.537 | 0.738 | 0.314 | 0.557 |
| 12 | 0.185 | 0.422 | 0.454 | 0.738 | 0.962 | 0.998 |
| 13 | 0.982 | 0.982 | 0.340 | 0.738 | 0.067 | 0.255 |
| 14 | 0.279 | 0.482 | 0.408 | 0.738 | 0.015 | 0.203 |
| 15 | 0.871 | 0.973 | 0.065 | 0.738 | 0.029 | 0.203 |
| 16 | 0.498 | 0.651 | 0.474 | 0.738 | 0.062 | 0.255 |
| 17 | 0.165 | 0.422 | 0.366 | 0.738 | 0.115 | 0.364 |
| 18 | 0.080 | 0.422 | 0.660 | 0.738 | 0.186 | 0.442 |
| 19 | 0.968 | 0.982 | 0.516 | 0.738 | 0.260 | 0.549 |

*Note.* *P*_fdr_ values are from two way mixed analyses of variance with Condition (cooperation vs competition) as the within subject factor and Group (GD vs HG vs HC) as the between subject factor. Multiple comparisons were controlled using FDR correction and effects with *P*_fdr_ smaller than 0.05 were considered significant.

**Table S7.** FDR corrected *p* values for group and condition effects

on channel to channel functional connectivity

| **Channel** | **Group_*P*** | **Group_*P*_fdr_** | **Condition_*P*** | **Condition_*P*_fdr_** | **Interaction_*P*** | **Interaction_*P*_fdr_** |
| --- | --- | --- | --- | --- | --- | --- |
| CH1-CH2 | 0.840 | 0.898 | 0.772 | 0.981 | 0.836 | 0.994 |
| CH1-CH3 | 0.397 | 0.617 | 0.295 | 0.954 | 0.506 | 0.994 |
| CH2-CH3 | 0.208 | 0.445 | 0.888 | 0.981 | 0.592 | 0.994 |
| CH1-CH4 | 0.263 | 0.484 | 0.282 | 0.954 | 0.057 | 0.994 |
| CH2-CH4 | 0.236 | 0.463 | 0.646 | 0.981 | 0.556 | 0.994 |
| CH3-CH4 | 0.714 | 0.838 | 0.895 | 0.981 | 0.452 | 0.994 |
| CH1-CH5 | 0.560 | 0.726 | 0.415 | 0.981 | 0.524 | 0.994 |
| CH2-CH5 | 0.362 | 0.583 | 0.889 | 0.981 | 0.558 | 0.994 |
| CH3-CH5 | 0.776 | 0.862 | 0.346 | 0.981 | 0.217 | 0.994 |
| CH4-CH5 | 0.433 | 0.638 | 0.564 | 0.981 | 0.213 | 0.994 |
| CH1-CH6 | 0.371 | 0.592 | 0.853 | 0.981 | 0.599 | 0.994 |
| CH2-CH6 | 0.225 | 0.463 | 0.059 | 0.849 | 0.826 | 0.994 |
| CH3-CH6 | 0.787 | 0.862 | 0.134 | 0.849 | 0.695 | 0.994 |
| CH4-CH6 | 0.060 | 0.279 | 0.428 | 0.981 | 0.040 | 0.994 |
| CH5-CH6 | 0.077 | 0.306 | 0.089 | 0.849 | 0.366 | 0.994 |
| CH1-CH7 | 0.789 | 0.862 | 0.384 | 0.981 | 0.467 | 0.994 |
| CH2-CH7 | 0.246 | 0.472 | 0.472 | 0.981 | 0.571 | 0.994 |
| CH3-CH7 | 0.872 | 0.915 | 0.045 | 0.849 | 0.835 | 0.994 |
| CH4-CH7 | 0.865 | 0.913 | 0.944 | 0.981 | 0.715 | 0.994 |
| CH5-CH7 | 0.074 | 0.306 | 0.747 | 0.981 | 0.817 | 0.994 |
| CH6-CH7 | 0.114 | 0.350 | 0.423 | 0.981 | 0.107 | 0.994 |
| CH1-CH8 | 0.224 | 0.463 | 0.875 | 0.981 | 0.108 | 0.994 |
| CH2-CH8 | 0.186 | 0.427 | 0.672 | 0.981 | 0.693 | 0.994 |
| CH3-CH8 | 0.454 | 0.647 | 0.465 | 0.981 | 0.639 | 0.994 |
| CH4-CH8 | 0.432 | 0.638 | 0.779 | 0.981 | 0.425 | 0.994 |
| CH5-CH8 | 0.023 | 0.220 | 0.576 | 0.981 | 0.326 | 0.994 |
| CH6-CH8 | 0.010 | 0.153 | 0.920 | 0.981 | 0.002 | 0.372 |
| CH7-CH8 | 0.012 | 0.166 | 0.947 | 0.981 | 0.517 | 0.994 |
| CH1-CH10 | 0.126 | 0.354 | 0.463 | 0.981 | 0.898 | 0.994 |
| CH2-CH10 | 0.054 | 0.279 | 0.518 | 0.981 | 0.232 | 0.994 |
| CH3-CH10 | 0.122 | 0.354 | 0.886 | 0.981 | 0.663 | 0.994 |
| CH4-CH10 | 0.238 | 0.463 | 0.205 | 0.954 | 0.059 | 0.994 |
| CH5-CH10 | 0.223 | 0.463 | 0.310 | 0.963 | 0.968 | 0.994 |
| CH6-CH10 | 0.581 | 0.737 | 0.071 | 0.849 | 0.896 | 0.994 |
| CH7-CH10 | 0.058 | 0.279 | 0.896 | 0.981 | 0.356 | 0.994 |
| CH8-CH10 | 0.378 | 0.599 | 0.438 | 0.981 | 0.927 | 0.994 |
| CH1-CH11 | 0.079 | 0.306 | 0.699 | 0.981 | 0.163 | 0.994 |
| CH2-CH11 | 0.577 | 0.736 | 0.288 | 0.954 | 0.792 | 0.994 |
| CH3-CH11 | 0.059 | 0.279 | 0.908 | 0.981 | 0.817 | 0.994 |
| CH4-CH11 | 0.339 | 0.552 | 0.352 | 0.981 | 0.919 | 0.994 |
| CH5-CH11 | 0.251 | 0.472 | 0.560 | 0.981 | 0.291 | 0.994 |
| CH6-CH11 | 0.559 | 0.726 | 0.111 | 0.849 | 0.929 | 0.994 |
| CH7-CH11 | 0.905 | 0.938 | 0.141 | 0.861 | 0.669 | 0.994 |
| CH8-CH11 | 0.134 | 0.370 | 0.680 | 0.981 | 0.603 | 0.994 |
| CH10-CH11 | 0.028 | 0.221 | 0.972 | 0.984 | 0.145 | 0.994 |
| CH1-CH12 | 0.072 | 0.306 | 0.107 | 0.849 | 0.552 | 0.994 |
| CH2-CH12 | 0.620 | 0.768 | 0.958 | 0.981 | 0.339 | 0.994 |
| CH3-CH12 | 0.111 | 0.350 | 0.464 | 0.981 | 0.342 | 0.994 |
| CH4-CH12 | 0.732 | 0.841 | 0.934 | 0.981 | 0.823 | 0.994 |
| CH5-CH12 | 0.039 | 0.268 | 0.751 | 0.981 | 0.826 | 0.994 |
| CH6-CH12 | 0.034 | 0.244 | 0.217 | 0.954 | 0.959 | 0.994 |
| CH7-CH12 | 0.483 | 0.668 | 0.177 | 0.954 | 0.909 | 0.994 |
| CH8-CH12 | 0.108 | 0.350 | 0.908 | 0.981 | 0.440 | 0.994 |
| CH10-CH12 | 0.248 | 0.472 | 0.234 | 0.954 | 0.190 | 0.994 |
| CH11-CH12 | 0.806 | 0.867 | 0.511 | 0.981 | 0.294 | 0.994 |
| CH1-CH13 | 0.091 | 0.321 | 0.198 | 0.954 | 0.970 | 0.994 |
| CH2-CH13 | 0.439 | 0.641 | 0.196 | 0.954 | 0.938 | 0.994 |
| CH3-CH13 | 0.690 | 0.831 | 0.129 | 0.849 | 0.931 | 0.994 |
| CH4-CH13 | 0.748 | 0.853 | 0.923 | 0.981 | 0.740 | 0.994 |
| CH5-CH13 | 0.593 | 0.744 | 0.808 | 0.981 | 0.204 | 0.994 |
| CH6-CH13 | 0.553 | 0.726 | 0.358 | 0.981 | 0.837 | 0.994 |
| CH7-CH13 | 0.926 | 0.950 | 0.916 | 0.981 | 0.986 | 0.994 |
| CH8-CH13 | 0.699 | 0.836 | 0.879 | 0.981 | 0.514 | 0.994 |
| CH10-CH13 | 0.023 | 0.220 | 0.441 | 0.981 | 0.782 | 0.994 |
| CH11-CH13 | 0.289 | 0.515 | 0.180 | 0.954 | 0.689 | 0.994 |
| CH12-CH13 | 0.126 | 0.354 | 0.168 | 0.954 | 0.228 | 0.994 |
| CH1-CH14 | 0.029 | 0.221 | 0.014 | 0.849 | 0.540 | 0.994 |
| CH2-CH14 | 0.454 | 0.647 | 0.355 | 0.981 | 0.638 | 0.994 |
| CH3-CH14 | 0.505 | 0.685 | 0.047 | 0.849 | 0.932 | 0.994 |
| CH4-CH14 | 0.759 | 0.860 | 0.602 | 0.981 | 0.921 | 0.994 |
| CH5-CH14 | 0.478 | 0.668 | 0.477 | 0.981 | 0.749 | 0.994 |
| CH6-CH14 | 0.931 | 0.950 | 0.024 | 0.849 | 0.883 | 0.994 |
| CH7-CH14 | 0.310 | 0.525 | 0.332 | 0.981 | 0.917 | 0.994 |
| CH8-CH14 | 0.332 | 0.545 | 0.131 | 0.849 | 0.693 | 0.994 |
| CH10-CH14 | 0.259 | 0.482 | 0.655 | 0.981 | 0.792 | 0.994 |
| CH11-CH14 | 0.116 | 0.350 | 0.604 | 0.981 | 0.432 | 0.994 |
| CH12-CH14 | 0.317 | 0.527 | 0.083 | 0.849 | 0.156 | 0.994 |
| CH13-CH14 | 0.934 | 0.950 | 0.645 | 0.981 | 0.607 | 0.994 |
| CH1-CH15 | 0.027 | 0.221 | 0.680 | 0.981 | 0.145 | 0.994 |
| CH2-CH15 | 0.952 | 0.963 | 0.270 | 0.954 | 0.737 | 0.994 |
| CH3-CH15 | 0.267 | 0.486 | 0.093 | 0.849 | 0.973 | 0.994 |
| CH4-CH15 | 0.732 | 0.841 | 0.794 | 0.981 | 0.904 | 0.994 |
| CH5-CH15 | 0.187 | 0.427 | 0.945 | 0.981 | 0.224 | 0.994 |
| CH6-CH15 | 0.108 | 0.350 | 0.079 | 0.849 | 0.699 | 0.994 |
| CH7-CH15 | 0.317 | 0.527 | 0.301 | 0.954 | 0.873 | 0.994 |
| CH8-CH15 | 0.021 | 0.220 | 0.718 | 0.981 | 0.954 | 0.994 |
| CH10-CH15 | 0.055 | 0.279 | 0.820 | 0.981 | 0.821 | 0.994 |
| CH11-CH15 | 0.096 | 0.328 | 0.681 | 0.981 | 0.985 | 0.994 |
| CH12-CH15 | 0.157 | 0.401 | 0.127 | 0.849 | 0.073 | 0.994 |
| CH13-CH15 | 0.193 | 0.434 | 0.563 | 0.981 | 0.682 | 0.994 |
| CH14-CH15 | 0.778 | 0.862 | 0.870 | 0.981 | 0.805 | 0.994 |
| CH1-CH16 | 0.002 | 0.095 | 0.200 | 0.954 | 0.551 | 0.994 |
| CH2-CH16 | 0.628 | 0.772 | 0.515 | 0.981 | 0.333 | 0.994 |
| CH3-CH16 | 0.571 | 0.735 | 0.398 | 0.981 | 0.545 | 0.994 |
| CH4-CH16 | 0.863 | 0.913 | 0.590 | 0.981 | 0.302 | 0.994 |
| CH5-CH16 | 0.183 | 0.427 | 0.920 | 0.981 | 0.164 | 0.994 |
| CH6-CH16 | 0.767 | 0.862 | 0.080 | 0.849 | 0.463 | 0.994 |
| CH7-CH16 | 0.391 | 0.613 | 0.760 | 0.981 | 0.713 | 0.994 |
| CH8-CH16 | 0.306 | 0.525 | 0.644 | 0.981 | 0.541 | 0.994 |
| CH10-CH16 | 0.058 | 0.279 | 0.218 | 0.954 | 0.970 | 0.994 |
| CH11-CH16 | 0.052 | 0.279 | 0.872 | 0.981 | 0.688 | 0.994 |
| CH12-CH16 | 0.185 | 0.427 | 0.297 | 0.954 | 0.313 | 0.994 |
| CH13-CH16 | 0.205 | 0.444 | 0.912 | 0.981 | 0.443 | 0.994 |
| CH14-CH16 | 0.175 | 0.427 | 0.549 | 0.981 | 0.657 | 0.994 |
| CH15-CH16 | 0.973 | 0.973 | 0.234 | 0.954 | 0.416 | 0.994 |
| CH1-CH17 | 0.048 | 0.279 | 0.850 | 0.981 | 0.099 | 0.994 |
| CH2-CH17 | 0.551 | 0.726 | 0.125 | 0.849 | 0.899 | 0.994 |
| CH3-CH17 | 0.089 | 0.321 | 0.101 | 0.849 | 0.618 | 0.994 |
| CH4-CH17 | 0.119 | 0.350 | 0.447 | 0.981 | 0.814 | 0.994 |
| CH5-CH17 | 0.198 | 0.440 | 0.473 | 0.981 | 0.531 | 0.994 |
| CH6-CH17 | 0.151 | 0.392 | 0.777 | 0.981 | 0.807 | 0.994 |
| CH7-CH17 | 0.066 | 0.296 | 0.845 | 0.981 | 0.884 | 0.994 |
| CH8-CH17 | 0.003 | 0.095 | 0.635 | 0.981 | 0.647 | 0.994 |
| CH10-CH17 | 0.021 | 0.220 | 0.822 | 0.981 | 0.596 | 0.994 |
| CH11-CH17 | 0.425 | 0.637 | 0.950 | 0.981 | 0.745 | 0.994 |
| CH12-CH17 | 0.173 | 0.427 | 0.171 | 0.954 | 0.099 | 0.994 |
| CH13-CH17 | 0.185 | 0.427 | 0.932 | 0.981 | 0.274 | 0.994 |
| CH14-CH17 | 0.878 | 0.916 | 0.272 | 0.954 | 0.630 | 0.994 |
| CH15-CH17 | 0.805 | 0.867 | 0.659 | 0.981 | 0.418 | 0.994 |
| CH16-CH17 | 0.792 | 0.862 | 0.950 | 0.981 | 0.419 | 0.994 |
| CH1-CH18 | 0.231 | 0.463 | 0.739 | 0.981 | 0.118 | 0.994 |
| CH2-CH18 | 0.402 | 0.619 | 0.648 | 0.981 | 0.497 | 0.994 |
| CH3-CH18 | 0.491 | 0.672 | 0.100 | 0.849 | 0.355 | 0.994 |
| CH4-CH18 | 0.658 | 0.798 | 0.575 | 0.981 | 0.833 | 0.994 |
| CH5-CH18 | 0.309 | 0.525 | 0.508 | 0.981 | 0.133 | 0.994 |
| CH6-CH18 | 0.484 | 0.668 | 0.686 | 0.981 | 0.653 | 0.994 |
| CH7-CH18 | 0.537 | 0.723 | 0.524 | 0.981 | 0.706 | 0.994 |
| CH8-CH18 | 0.056 | 0.279 | 0.625 | 0.981 | 0.860 | 0.994 |
| CH10-CH18 | 0.092 | 0.321 | 0.360 | 0.981 | 0.656 | 0.994 |
| CH11-CH18 | 0.077 | 0.306 | 0.977 | 0.984 | 0.827 | 0.994 |
| CH12-CH18 | 0.141 | 0.384 | 0.046 | 0.849 | 0.193 | 0.994 |
| CH13-CH18 | 0.408 | 0.623 | 0.955 | 0.981 | 0.510 | 0.994 |
| CH14-CH18 | 0.724 | 0.841 | 0.804 | 0.981 | 0.587 | 0.994 |
| CH15-CH18 | 0.273 | 0.491 | 0.671 | 0.981 | 0.434 | 0.994 |
| CH16-CH18 | 0.559 | 0.726 | 0.978 | 0.984 | 0.265 | 0.994 |
| CH17-CH18 | 0.019 | 0.220 | 0.630 | 0.981 | 0.239 | 0.994 |
| CH1-CH19 | 0.596 | 0.744 | 0.556 | 0.981 | 0.098 | 0.994 |
| CH2-CH19 | 0.111 | 0.350 | 0.769 | 0.981 | 0.790 | 0.994 |
| CH3-CH19 | 0.178 | 0.427 | 0.125 | 0.849 | 0.879 | 0.994 |
| CH4-CH19 | 0.148 | 0.392 | 0.527 | 0.981 | 0.998 | 0.998 |
| CH5-CH19 | 0.080 | 0.306 | 0.387 | 0.981 | 0.676 | 0.994 |
| CH6-CH19 | 0.006 | 0.109 | 0.994 | 0.994 | 0.925 | 0.994 |
| CH7-CH19 | 0.044 | 0.279 | 0.709 | 0.981 | 0.762 | 0.994 |
| CH8-CH19 | 0.003 | 0.095 | 0.331 | 0.981 | 0.989 | 0.994 |
| CH10-CH19 | 0.030 | 0.221 | 0.740 | 0.981 | 0.695 | 0.994 |
| CH11-CH19 | 0.202 | 0.442 | 0.687 | 0.981 | 0.935 | 0.994 |
| CH12-CH19 | 0.030 | 0.221 | 0.285 | 0.954 | 0.151 | 0.994 |
| CH13-CH19 | 0.420 | 0.636 | 0.873 | 0.981 | 0.910 | 0.994 |
| CH14-CH19 | 0.471 | 0.665 | 0.116 | 0.849 | 0.574 | 0.994 |
| CH15-CH19 | 0.304 | 0.525 | 0.457 | 0.981 | 0.663 | 0.994 |
| CH16-CH19 | 0.231 | 0.463 | 0.375 | 0.981 | 0.467 | 0.994 |
| CH17-CH19 | 0.709 | 0.838 | 0.377 | 0.981 | 0.613 | 0.994 |
| CH18-CH19 | 0.001 | 0.095 | 0.806 | 0.981 | 0.854 | 0.994 |
| CH1-CH9 | 0.964 | 0.970 | 0.931 | 0.981 | 0.126 | 0.994 |
| CH2-CH9 | 0.715 | 0.838 | 0.755 | 0.981 | 0.790 | 0.994 |
| CH3-CH9 | 0.118 | 0.350 | 0.096 | 0.849 | 0.624 | 0.994 |
| CH4-CH9 | 0.149 | 0.392 | 0.269 | 0.954 | 0.509 | 0.994 |
| CH5-CH9 | 0.004 | 0.095 | 0.219 | 0.954 | 0.179 | 0.994 |
| CH6-CH9 | 0.053 | 0.279 | 0.614 | 0.981 | 0.210 | 0.994 |
| CH7-CH9 | 0.000 | 0.020 | 0.099 | 0.849 | 0.231 | 0.994 |
| CH9-CH10 | 0.057 | 0.279 | 0.006 | 0.849 | 0.652 | 0.994 |
| CH9-CH11 | 0.237 | 0.463 | 0.265 | 0.954 | 0.114 | 0.994 |
| CH9-CH12 | 0.072 | 0.306 | 0.852 | 0.981 | 0.851 | 0.994 |
| CH9-CH13 | 0.292 | 0.515 | 0.532 | 0.981 | 0.650 | 0.994 |
| CH9-CH14 | 0.449 | 0.647 | 0.019 | 0.849 | 0.442 | 0.994 |
| CH9-CH15 | 0.004 | 0.095 | 0.274 | 0.954 | 0.662 | 0.994 |
| CH9-CH16 | 0.645 | 0.788 | 0.237 | 0.954 | 0.235 | 0.994 |
| CH9-CH17 | 0.006 | 0.109 | 0.702 | 0.981 | 0.329 | 0.994 |
| CH9-CH18 | 0.088 | 0.321 | 0.769 | 0.981 | 0.538 | 0.994 |
| CH9-CH19 | 0.004 | 0.095 | 0.907 | 0.981 | 0.518 | 0.994 |
| CH8-CH9 | 0.014 | 0.180 | 0.299 | 0.954 | 0.688 | 0.994 |

*Note.* *P*_fdr_ values are from two way mixed analyses of variance with Condition (cooperation vs competition) as the within subject factor and Group (GD vs HG vs HC) as the between subject factor. Multiple comparisons were controlled using FDR correction and effects with *P*_fdr_ smaller than 0.05 were considered significant.


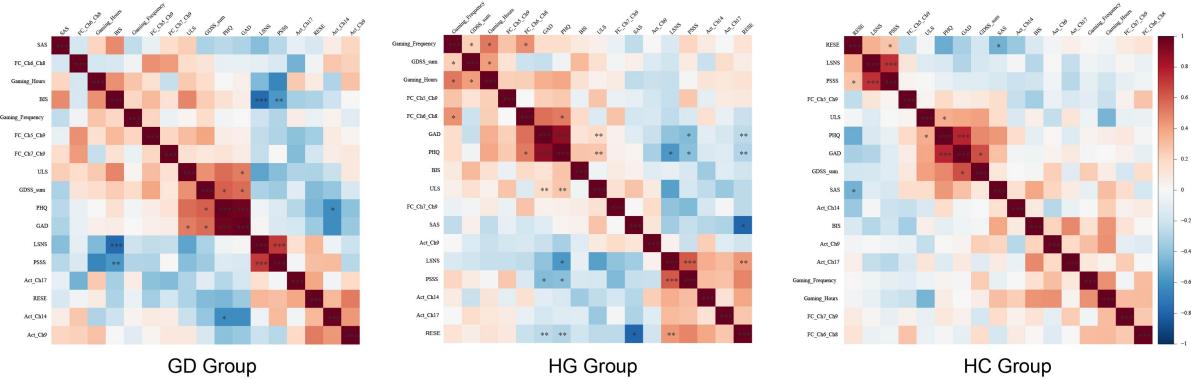


**Fig. S1. Correlation heatmaps between fNIRS measures and behavioral performance.** Each panel shows correlations between cortical activation or functional connectivity and behavioral scale scores across the three groups. Red indicates positive correlations; blue indicates negative correlations. ^*^*p* < 0.05, ^**^*p* < 0.01, ^***^*p* < 0.001.

**References:**

Alorani, O. I., & Alradaydeh, M. T. F. (2018). Spiritual well-being, perceived social support, and life satisfaction among university students. *International Journal of Adolescence and Youth, 23*(3), 291-298.

Cui, X., Bryant, D. M., & Reiss, A. L. (2012). NIRS-based hyperscanning reveals increased interpersonal coherence in superior frontal cortex during cooperation. *Neuroimage, 59*(3), 2430-2437.

Du, H., King, R. B., & Chi, P. (2012). The development and validation of the Relational Self‐Esteem Scale. *Scandinavian Journal of Psychology, 53*(3), 258-264.

Ip, H., Suen, Y. N., Hui, C. L. M., Wong, S. M. Y., Chan, S. K. W., Lee, E. H. M., ... & Chen, E. Y. H. (2022). Assessing anxiety among adolescents in Hong Kong: psychometric properties and validity of the Generalised Anxiety Disorder-7 (GAD-7) in an epidemiological community sample. *BMC psychiatry, 22*(1), 703.

Lubben, J., & Gironda, M. (2003). Centrality of social ties to the health and well-being of older adults. *Social work and health care in an aging world*, 319-350.

Levis, B., Benedetti, A., & Thombs, B. D. (2019). Accuracy of Patient Health Questionnaire-9 (PHQ-9) for screening to detect major depression: individual participant data meta-analysis. *bmj, 365*.

Novotny, P. J., Smith, D. J., Guse, L., Rummans, T. A., Hartmann, L., Alberts, S., ... & Sloan, J. A. (2010). A pilot study assessing social support among cancer patients enrolled on clinical trials: a comparison of younger versus older adults. *Cancer Management and Research*, 133-142.

Song, Q., Zheng, K., Ding, Z., Miao, Z., Liu, Z., Cheng, M., & Yi, J. (2024). Psychometric Properties of Different Short Forms of Social Interaction Anxiety Scale in Chinese College Students. *Psychology Research and Behavior Management,* 3405-3418.

Vasconcelos, A. G., Malloy-Diniz, L., & Correa, H. (2012). Systematic review of psychometric proprieties of Barratt Impulsiveness Scale Version 11 (BIS-11). *Clinical Neuropsychiatry, 9*(2).

Watabe, M., Kato, T. A., Teo, A. R., Horikawa, H., Tateno, M., Hayakawa, K., ... & Kanba, S. (2015). Relationship between trusting behaviors and psychometrics associated with social network and depression among young generation: A pilot study. *PLoS One, 10*(4), e0120183.

Winch, R. F. (1965). Rosenberg: society and the adolescent self-image (book review). *Social forces, 44*(2), 255.

Wu, C. H., & Yao, G. (2008). Psychometric analysis of the short-form UCLA Loneliness Scale (ULS-8) in Taiwanese undergraduate students. *Personality and Individual Differences, 44*(8), 1762-1771.

Yildiz, M. A., & Duy, B. (2014). Adaptation of the short-form of the UCLA loneliness scale (ULS-8) to Turkish for the adolescents. *Dusunen Adam: Journal of Psychiatry & Neurological Sciences, 27*(3).

Zimet, G. D., Dahlem, N. W., Zimet, S. G., & Farley, G. K. (1987). Perceived social support scale. *Psychosomatic Medicine*.

Zsido, A. N., Varadi-Borbas, B., & Arato, N. (2021). Psychometric properties of the social interaction anxiety scale and the social phobia scale in Hungarian adults and adolescents. *BMC psychiatry, 21*(1), 171.
