# Supplementary figures and images for: Disrupted brain functional networks in adolescents and young adults with gaming disorder during social interaction: An fNIRS study
Source: Psychol Med. 2026 Jun 8;56:e189. doi: 10.1017/S0033291726104176 (PMC13247798; doi:10.1017/S0033291726104176)

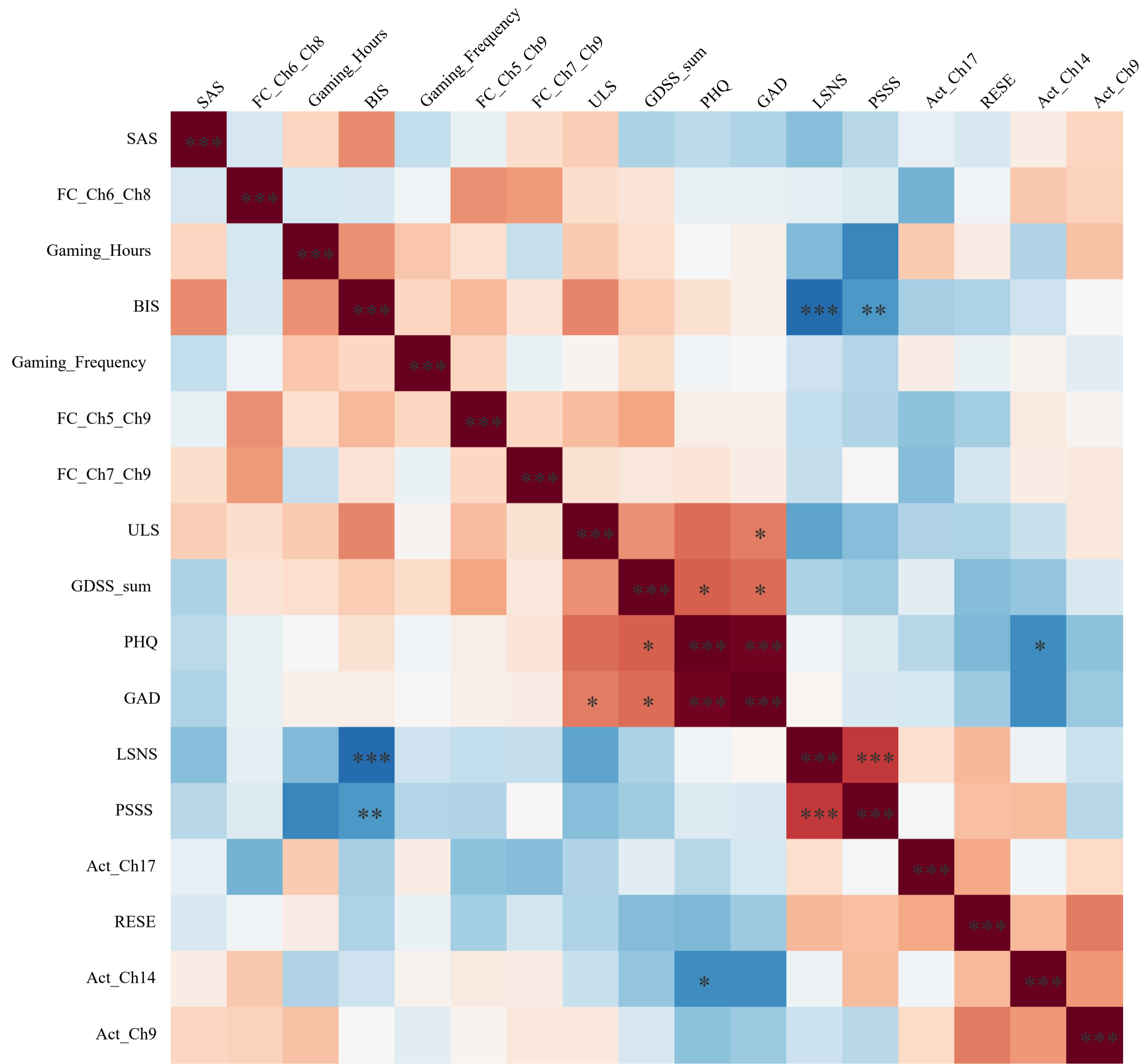

GD Group

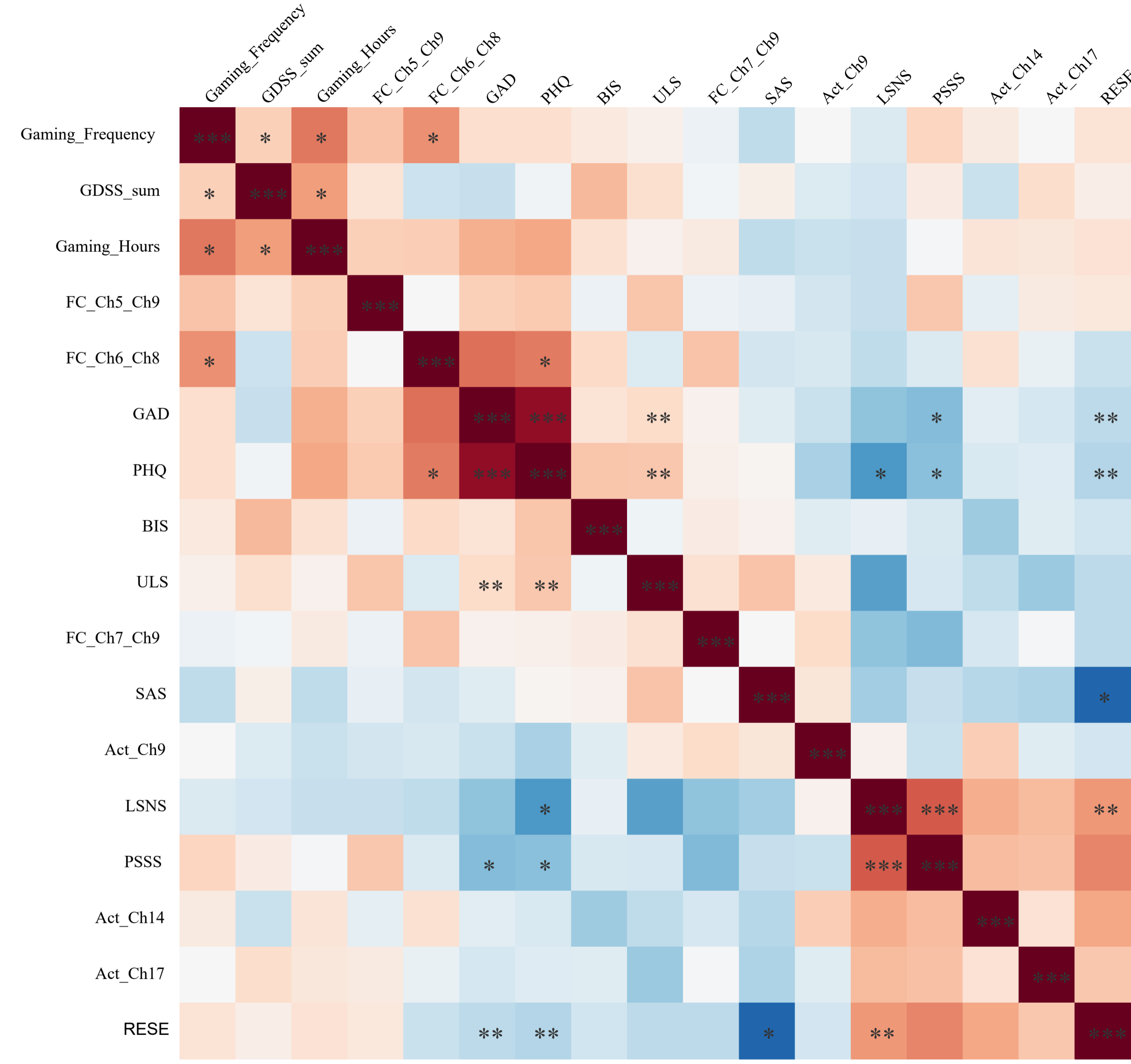

HG Group

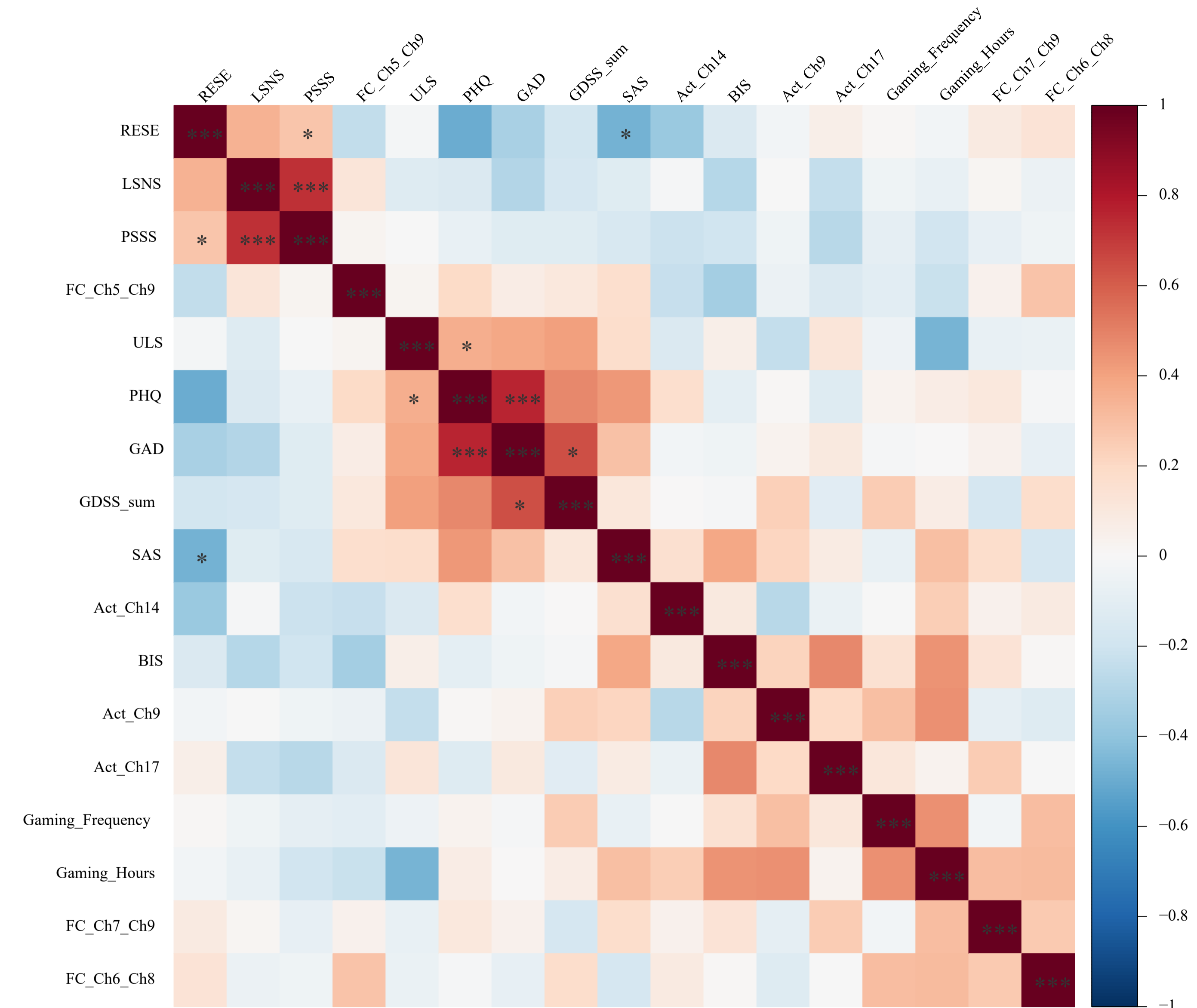

HC Group

Supplement: Wang et al. supplementary material 2 — Wang et al. supplementary material [file S0033291726104176sup002.pdf]
